# Mir-21 Suppression Promotes Mouse Hepatocarcinogenesis

**DOI:** 10.3390/cancers13194983

**Published:** 2021-10-04

**Authors:** Marta Correia de Sousa, Nicolas Calo, Cyril Sobolewski, Monika Gjorgjieva, Sophie Clément, Christine Maeder, Dobrochna Dolicka, Margot Fournier, Laurent Vinet, Xavier Montet, Jean-François Dufour, Bostjan Humar, Francesco Negro, Christine Sempoux, Michelangelo Foti

**Affiliations:** 1 Department of Cell Physiology and Metabolism, Faculty of Medicine, University of Geneva, 1211 Geneva, Switzerland; Marta.Sousa@unige.ch (M.C.d.S.); nicolas.calo@unige.ch (N.C.); Cyril.Sobolewski@unige.ch (C.S.); Monika.Gjorgjieva@unige.ch (M.G.); christine.maeder@unige.ch (C.M.); Dobrochna.Dolicka@unige.ch (D.D.); margot.fournier@unige.ch (M.F.); 2 Division of Clinical Pathology, Geneva University Hospitals, 1206 Geneva, Switzerland; Sophie.Clement@unige.ch (S.C.); Francesco.Negro@unige.ch (F.N.); 3 Department of Radiology, Faculty of Medicine, University of Geneva, 1206 Geneva, Switzerland; Laurent.vinet@unige.ch (L.V.); xmontet@infomaniak.ch (X.M.); 4 Department for Visceral Surgery and Medicine, University Hospital Bern, 3010 Bern, Switzerland; jf.dufour@svmed.ch; 5 Department of Visceral & Transplantation Surgery, University Hospital Zürich, 8006 Zürich, Switzerland; bostjan.humar@usz.ch; 6 Service of Clinical Pathology, University Institute of Pathology, Vaud University Hospital Center, 1011 Lausanne, Switzerland; Christine.Sempoux@chuv.ch

**Keywords:** microRNA 21, HCC, oncogenes, tumor suppressors, inflammation, fibrosis, immune cells, PTEN

## Abstract

**Simple Summary:**

Hepatocellular carcinoma (HCC) is a frequent cancer of the liver with limited therapeutic options. MicroRNAs are a class of small molecules regulating a wide range of cellular processes that are important for cancer development. Among these microRNAs, miR-21 is strongly upregulated in almost all human cancers including HCC, and is considered as a strong driver of cancer development, suggesting that its pharmacological inhibition might represent a potential therapy. In this study, we show that deletion of miR-21 in genetically engineered mice promotes instead the development of HCC in several mouse models of this liver cancer. We further show that the lack of miR-21 is associated with increases in the expression of oncogenes such as *Cdc25a*, subtle deregulations of the MAPK, HiPPO, and STAT3 signaling pathways, as well as alterations of the inflammatory/immune anti-tumoral responses in the liver, which overtime contribute to enhanced tumorigenesis and progression toward malignancy. These results call for cautiousness when considering miR-21 inhibition for therapeutic purposes in HCC.

**Abstract:**

The microRNA 21 (miR-21) is upregulated in almost all known human cancers and is considered a highly potent oncogene and potential therapeutic target for cancer treatment. In the liver, miR-21 was reported to promote hepatic steatosis and inflammation, but whether miR-21 also drives hepatocarcinogenesis remains poorly investigated in vivo. Here we show using both carcinogen (Diethylnitrosamine, DEN) or genetically (PTEN deficiency)-induced mouse models of hepatocellular carcinoma (HCC), total or hepatocyte-specific genetic deletion of this microRNA fosters HCC development—contrasting the expected oncogenic role of miR-21. Gene and protein expression analyses of mouse liver tissues further indicate that total or hepatocyte-specific miR-21 deficiency is associated with an increased expression of oncogenes such as *Cdc25a*, subtle deregulations of the MAPK, HiPPO, and STAT3 signaling pathways, as well as alterations of the inflammatory/immune anti-tumoral responses in the liver. Together, our data show that miR-21 deficiency promotes a pro-tumoral microenvironment, which over time fosters HCC development via pleiotropic and complex mechanisms. These results question the current dogma of miR-21 being a potent oncomiR in the liver and call for cautiousness when considering miR-21 inhibition for therapeutic purposes in HCC.

## 1. Introduction

Hepatocellular carcinoma (HCC) is the most frequent primary liver cancer and represents the fourth most deadly cancer [1]. HCC mostly develops in a context of chronic liver injury, such as sustained inflammation/fibrosis and cirrhosis [2]. Viral infection (hepatitis B or C) and alcoholic liver disease are currently the major risk factors for HCC development. However, increasing evidence indicates that non-alcoholic fatty liver disease (NAFLD), with or without cirrhosis, is likewise becoming a major etiological driver of liver cancer [1,3].

Besides well-known mutations associated with HCC (e.g., *TERT*, *TP53*, *MYC*, *WNT*, *CTNNB1*, *CCND1*, *PTEN*, [1,2]), numerous other mechanisms are deregulated and contribute to the development and heterogeneity of HCC, including DNA methylation events, chromatin structure changes, and post-transcriptional regulation of cancer-related factors by non-coding RNAs (e.g., microRNAs (miRNAs), long non-coding RNAs), or RNA-binding proteins [4,5,6,7]. Akin to genetic mutations, these non-genomic alterations can strongly affect the tumor microenvironment, the immune response, and the expression/activity of cancer-related factors (e.g., tumor suppressors or oncogenes), thereby markedly affecting disease development [1,8]. The diversity of mutations and other mechanisms fostering HCC development is reflected in the high degree of heterogeneity at the histological and molecular level within tumoral nodules even of the same patient, and/or depending on the etiological factors/genetic background [1,8,9]. The histological and molecular heterogeneity requires that treatment of HCC must be individually adjusted [10]. Liver resection and transplantation, often preceded by transarterial chemoembolization or radiofrequency ablations, are currently the only efficient curative treatments, but are limited to eligible patients having only early-stage diseases and non-metastatic tumors [11]. Patients with advanced disease, non-resectable tumors, or metastases are treated with systemic chemotherapy (e.g., Sorafenib, lenvatinib) but these drugs are poorly effective and are associated with serious side effects [12,13]. Unraveling druggable molecular mechanisms associated with HCC is therefore of key importance for developing new and alternative therapies for this cancer [2].

In this regard, the targeting of microRNAs has recently emerged as a promising therapeutic strategy for a wide range of pathologies, including cancer [14,15]. These small non-coding RNAs are post-transcriptional regulators of gene expression. They act through degradation or translational repression of messenger RNAs (mRNAs) by binding to specific regions, i.e., miRNA-responsive elements (MREs), mostly located in the 3’UTR of the transcripts [16,17]. Importantly, one miRNA can target hundreds of different transcripts, therefore being capable of modulating various cellular pathways simultaneously [18]. Alterations in miRNA expression/activity occur in HCC, but also already in chronic liver diseases preceding HCC (e.g., NAFLD/NASH, viral infections, alcoholic liver disease) with association to both hepatic disease development and malignant transformation [6,19].

Among these miRNAs, miR-21 has been extensively investigated in different cancers where it was shown to target important tumor suppressors such as, for example, *PTEN, SPRY2, TIMP3, PDCD4, RECK*, and *STAT3*, and thus to behave as one of the most important oncogenic miRNAs (oncomiR) identified to date [20]. In the liver, miR-21 expression/activity was repeatedly found to be increased in patients diagnosed with chronic hepatic diseases and murine models of NAFLD and NASH [21,22,23,24,25], suggesting that it might represent a potential therapeutic target [6,20,21,26]. In this regard, we and others previously reported that genetic deletion of miR-21 in vivo counteracted the development of steatosis and inflammation in different conditions [22,24,27]. Overexpression of miR-21 is also observed in human primary HCC and hepatic cancer cell lines [28], and its expression positively correlates with tumor dedifferentiation and poor life expectancy in patients [29]. In vitro approaches using established HCC cell lines or as xenografts in mice pointed to a pro-tumorigenic effect of miR-21 on proliferation, migration, and invasiveness [26,28,30,31,32,33]. In primary mouse hepatocytes, miR-21 was also reported to induce cell proliferation and epithelial-to-mesenchymal transition [34,35]. However, only a few studies investigated the in vivo role of miR-21 in orthotopic HCC models and these led to discrepant conclusions. Indeed, while injection of synthetic miR-21 inhibitors (antagomiRs) was suggested to decrease tumor occurrence and growth in hepatocyte-specific PTEN knockout mice (LPTENKO) [36], both miR-21 antagomiRs or miR-21 genetic deletion did not affect HCC development in adult mice following injection of high doses of diethylnitrosamine (DEN) and CCl4 [37].

To clarify the potential oncogenic role of miR-21 in HCC development, we investigated herein the impact of miR-21 total (miR21KO mice) and/or hepatocyte-specific (LmiR21KO mice) genetic deletion in HCC development induced over one year after a single DEN injection, and the impact of hepatocyte-specific simultaneous deletion of the tumor suppressor PTEN and miR-21 (LPTENmiR21KO mice). Strikingly and unexpectedly, our data indicate that miR-21 deficiency fosters hepatic tumorigenesis suggesting a protective role of this microRNA in HCC development.

## 2. Materials and Methods

### 2.1. Animals

Constitutive total miR-21 knockout mice (*Ddx4-Cre; miR21^lox/lox^*, miR21KO) and hepatocyte-specific miR-21 knockout mice (*Alb-Cre; miR21^lox/lox^*, LmiR21KO) were previously described [22]. Mice with a hepatocyte-specific deletion of both *PTEN* and *miR-21a* were generated by backcrossing hepatocyte-specific *Pten* knockout mice (*Alb-Cre; Pten^lox/lox^* [38,39]) with LmiR21KO mice. *Mir21^lox/lox^* mice were used as controls (CTRL—miR21fx) for miR21KO and LmiR21KO mice, while mice with floxed alleles but not carrying the *Alb-Cre* recombinase gene were used as controls (CTRL) for LPTENKO and LPTENmiR21KO. Silencing of the floxed alleles was confirmed by conventional PCR-based genotyping and real-time PCR for mature miR-21 and floxed exons 4 and 5 of the *Pten* gene. All animals had a mixed genetic background (C57BL/6Ncr, 129S6) and were housed in standard conditions in a conventional animal facility (groups of 4–5 individuals per cage, a 12 h/12 h light/dark cycle, ad libitum water, and standard chow diet (SDS RM3)). LmiR21KO mice were challenged with a high-fat diet (HFD) for 3 weeks, as previously described [22] before RNA sequencing (Rnaseq) analyses of liver tissues. All experiments were ethically approved by the Geneva Health head office and in accordance with the Swiss guidelines for animal experimentation. 

For the carcinogen-induced HCC model, DEN (25 mg/kg of body weight, Sigma-Aldrich) was injected intraperitoneally in 15-day-old animals, which were sacrificed 11 months post-birth. For the acute DEN-injection model, 2-month-old animals from each group were injected with 100 mg/kg of body weight and sacrificed 48 h post-injection, as previously described [40]. LPTENKO, LPTENmiR21KO, and corresponding CTRL mice were sacrificed at 7 and 12 months of age. Blood and liver/tumor samples were collected for histology and/or snap-frozen in liquid nitrogen and stored at −80 °C. Prior to storage, tumors isolated from sacrificed animals were counted and measured.

### 2.2. RNA Sequencing Analysis

RNA was extracted for high throughput sequencing from liver samples of LmiR21KO and CTRL mice challenged with HFD for three weeks, as previously described [22]. Poly-A selected RNA was used for library preparation with the TruSeq RNA Sample preparation kit (Illumina), according to the manufacturer’s protocol, and submitted to 100 nt single read sequencing in an Illumina HiSeq 2500 system.

Reads were aligned using TopHat v.1.3.3 software (mm9 release) and further processed with RSeQC-2.3.3, PicardTools v.1.88, and HTSeq v0.5.3p9 for biological quality control and preparation of table of counts. Normalization and differential expression analysis were performed using the R/Bioconductor DESeq package (v.1.9.14). Raw count data were normalized by the library size and differentially expressed genes were estimated with the negative binomial general model statistics. Morpheus software (https://software.broadinstitute.org/morpheus, accessed on 25 March 2021) was used to visualize log10-transformed normalized data and perform hierarchical clustering. Differentially expressed genes were selected by log10FC (<−0.05; >0.05) and *p*-value <0.05. Validation of RNAseq data was then performed by reassessing the expression of top candidate genes of interest identified by our in silico analyses of the RNAseq data in the LmiR21KO and CTRL mice challenged with HFD. Data indicate that >96% of the genes reanalyzed by RT-qPCR display similar expression profiles in miR-21 deficient mice as those uncovered by RNAseq analyses (Figure S1C). 

### 2.3. Histology

Fresh liver and tumor samples were fixed overnight in 4% paraformaldehyde solution and washed with PBS. Following dehydration and paraffin-embedding, 5 µm tissue sections were stained with hematoxylin/eosin (H&E) for morphological analysis or trichrome Masson for fibrosis investigation. Images were acquired using an Olympus VS120 slide scanner (Olympus, Tokyo, Japan) at a 40× magnification and sent for blind analysis to a pathologist specialized in liver pathology (Prof. Christine Sempoux, Lausanne, Switzerland) to evaluate the presence or absence of steatosis, inflammatory foci, and fibrosis, as well as to characterize the type tumoral nodules present in mouse livers.

### 2.4. Immunohistochemistry

Four-micrometer sections of formalin-fixed and paraffin-embedded liver biopsy specimens were deparaffinized with xylene and rehydrated with decreasing concentrations of ethanol in water. Antigen retrieval was achieved in sodium citrate buffer (pH 6.0) for 20 min at 95 °C. Tissues were stained by immunohistochemistry with either anti-CD8a antibody (1:5000, Ebioscience, ThermoFisher Scientific, San Diego, CA, USA, clone 4SM15, catalog number: 14-0808-82), anti-CD4 (1:400, Cell signaling, Danvers, MA, USA, clone D7D22, catalog number: #25229), anti-CD3 (1:1000, Dako, Agilent, Santa Clara, CA, USA, clone A0452, catalog number: 790-4341), or anti-Ki67 antibody (1:150, Millipore, Merk, Darmstadt, Germany, catalog number: AB9260). The reaction was revealed using EnVision™+System-HRP (Dako, Agilent, catalog number: K4003) or DAB Substrate Kit (Abcam, Cambridge, UK, catalog number: ab64238), and sections were counterstained with hematoxylin. Images were acquired using a panoramic 250 Flash III digital slide scanner (3DHistech, Budapest, Hungary) or with AxioScan.Z1 (Carl Zeiss Microscopy GmbH, Jena, Germany) with a 20× objective and analyzed using Definiens System (Definiens AG, Munich, Germany) or ZEN2 blue edition software (Carl Zeiss Microscopy GmbH, Jena, Germany).

### 2.5. Magnetic Resonance Imaging (MRI) and CT-Scan Imaging (CT)

CTRL, miR21KO, and LmiR21KO mice injected with DEN were scanned using EchoMRI-700 quantitative nuclear magnetic resonance analyzer (Echo Medical Systems, Houston, HX, USA) following retro-orbital injection of Primovist^®^ (Bayer Healthcare, Berlin, Germany) contrasting agent diluted 1:40 in 0.9% NaCl. Image analysis for quantification of tumor number, size, and volume was performed using OsiriX MD v.10.0.1 software (OsiriX MD, Geneva, Switzerland).

Preceding sacrifice, CTRL, LPTENKO, and LPTENmiR21KO mice were injected retro-orbitally with 100 µL ExiTron nano 6000 (catalog number: 130-095-147, Viscover, Berlin, Germany) diluted 1:1 in 0.9% NaCl and scanned using a Quantum GX microCT (PerkinElmer, Waltham, MA, USA). Image analysis for three-dimensional reconstruction and quantification of tumor number, size, and volume was performed using OsiriX MD v.10.0.1 (OsiriX MD, Geneva, Switzerland). 

### 2.6. Real-Time PCR

Total RNA was extracted using TRIzol reagent (catalog number: 15596026, Invitrogen, Thermo Fisher Scientific, Carlsbad, CA, USA) and cDNA synthesis was performed using 1 ug RNA and High-Capacity RNA-to-cDNA kit (catalog number: 4388950, Applied Biosystems™, Thermo Fisher Scientific, Carlsbad, CA), according to the manufacturer’s instructions. Real-time PCR was performed using PowerUp™ SYBR™ Green Master Mix (catalog number: A25743, Applied Biosystems ™, Thermo Fisher Scientific, Carlsbad, CA, USA) on QuantStudio™ systems with respective software (Applied Biosystems™, Thermo Fisher Scientific, Carlsbad, CA, USA). Gene expression was normalized with *CypA*, *Rps9*, *Gak*, and *Srp72* as housekeeping genes by using geNorm application [41]. Primer sequences are described in Table S1.

### 2.7. Western Blot

Proteins from mouse hepatic tissues were extracted and lysed in RIPA buffer as described previously [22]. The proteins were separated in a 5–20% SDS gradient gel electrophoresis and were further transferred to a nitrocellulose membrane via a wet transfer system. Saturation of membranes was done with polyvinyl alcohol (PVA) or TBS-Tween 0.1%-milk-5%. Membranes were then washed with TBS-Tween 0.1%, and incubated with the appropriate primary antibody overnight, diluted in TBS-Tween 0.1%-milk-5% or in TBS-Tween 0.1%-BSA-3–5% following the manufacturer’s instructions. After overnight incubation at 4 °C, membranes were washed with TBS-Tween 0.1% and incubated with the secondary antibody conjugated with horseradish peroxidase (HRP) for 1 h at room temperature. Detection of proteins was done using a chemiluminescent detection reagent (Amersham™ ECL, catalog number: RPN2232, Cytiva Life Sciences, Marlborough, MA, USA). Blots were imaged with the Syngene PXi technology, and the bands were quantified using the GeneTools from Syngene software (4.3.9.0, Synoptics, Cambridge, UK). Antibodies used in this study and corresponding dilutions are listed in Table S2.

### 2.8. Cell Culture

Human hepatic cancer cell lines (HepG2 and Huh7) were cultured in DMEM medium (21885025, Gibco™, Thermo Fisher Scientific, Grand Island, NY, USA) supplemented with fetal bovine serum (10%). A total of 24 h after seeding, cells were transfected with 20 nM of miR-21-5p mimicking oligonucleotides (miRIDIAN, Horizon Discovery, Cambridge, UK, ref. C-300492-03-0002) or miR-21-5p antagomiRs (miRIDIAN, Horizon Discovery, Cambridge, UK, ref. IH-300492-05-0002) and respective control nucleotides, using the transfection agent Interferin (catalog number: 409-10, Polyplus-transfection, Illkirch, France). After 48 h of transfection, cells were detached, and RNA was isolated and processed as described in Section 2.6.

### 2.9. In Silico Analysis

#### 2.9.1. List of Predicted and Validated Targets

Predicted and validated targets of miR-21-5p and miR-21-3p were retrieved using the miRWalk database (http://mirwalk.umm.uni-heidelberg.de/, accessed date: 11 June 2020) [42]. Only targets predicted by at least four different algorithms (miRWalk, miRanda, RNA22, and Targetscan,) were considered for Gene Ontology (GO) enrichment analysis.

#### 2.9.2. Gene Ontology Enrichment Analysis

GO enrichment analysis of deregulated genes identified in liver-inducible miR-21 knockout mice challenged with HFD was performed using DAVID database with functional categorization by biological processes and a *p*-value cut-off for false discovery rate (FDR) of 0.05 (https://david.ncifcrf.gov/, accessed on 27 March 2020) [43,44].

#### 2.9.3. Identification of Genes of Potential Interest and Related Biological Functions

The list of predicted/validated miR-21 targets was cross-referenced with deregulated genes identified through the transcriptomic analysis of liver-specific miR-21 knockout mice challenged with HFD (*p*-value cut-off = 0.05; |log2FC| > 0.5). The 124 candidate genes identified through this cross-referencing were then processed through the CancerMine database [45] to classify them as oncogenes, drivers, or tumor suppressors (accessed on 07/06/2021). Genes potentially upregulated by miR-21 suppression were finally considered for GO enrichment analysis by a biological process with term-fusion using ClueGO+CluePedia application (v2.5.8) [46,47] in Cytoscape software (v3.8.2, https://cytoscape.org/ accessed on 22 June 2021) [48] and by KEGG pathways using shinyGOv0.61 application (http://bioinformatics.sdstate.edu/go/, accessed on 22 June 2021) [49].

### 2.10. Statistical Analysis

Statistical analyses were performed using the GraphPad Prism 8 software (GraphPad Software, San Diego, CA, USA). Results are expressed as mean ± standard deviation (SD). The outliers test was performed using the ROUT method (Q = 1%). For comparison of two groups, an unpaired *t*-test with Welch’s correction was used. For comparison of more than two groups, a one-way ANOVA test followed by Holm-Sidak correction was performed. To evaluate the independence of categorical variables, the chi-square test was used. Results of statistical tests are indicated in the figures as follows: * *p* < 0.05, ** *p* < 0.01, *** *p* < 0.001 or, **** *p* < 0.0001.

## 3. Results

### 3.1. miR-21 Suppression in the Liver Deregulates Numerous Genes Relevant for Hepatocarcinogenesis and Immune Responses

**Figure 1:**
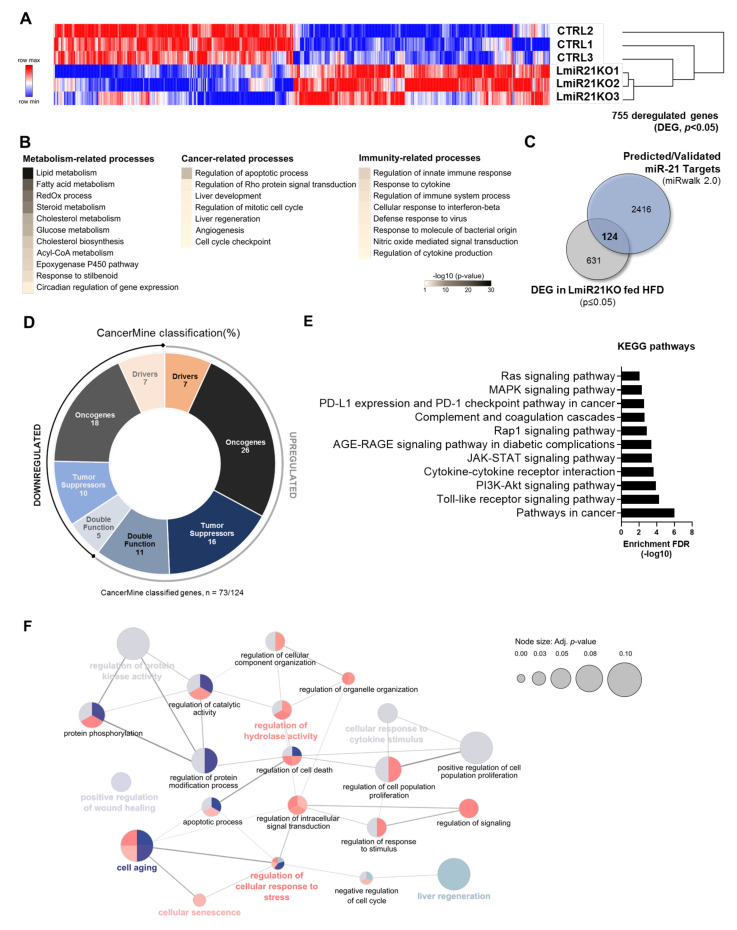
Transcriptomic profile of cancer-related factors altered in hepatic tissues from LmiR-21KO livers. (**A**) Heatmap representation of differentially expressed genes (DEG, *p*-value < 0.05, |log10FC| > 0.5) identified through RNAseq analysis of explanted liver tissues from control (CTRL) and hepatocyte-specific miR-21 knockout mice fed obesogenic diet (LmiR21KO, HFD 60% of fat, high-fat diet) for 3 weeks (*n* = 3 per group, upregulated and downregulated genes represented in red and blue, respectively). (**B**) Gene Ontology (GO) enrichment analysis of DEG using DAVID database ((https://david.ncifcrf.gov/, accessed on 27 March 2020) retrieved metabolism-, cancer-, and immunity-related processes. (**C**) Potential miR-21 targets of interest were identified by cross-comparison of DEG identified in LmiR21KO with a list of predicted/validated targets of miR-21-5p/-3p extracted from the miRwalk 2.0 database (accessed on 11 June 2020). (**D**) Literature screening and the CancerMine database (accessed on 07/06/2021) identified candidates as oncogenes (ONC—black and dark grey for upregulated and downregulated genes, respectively), tumor suppressors (TS—dark blue and light blue for upregulated and downregulated genes, respectively), or drivers (D—orange and light orange for upregulated and downregulated genes, respectively) of carcinogenesis. Genes identified as both ONC and TS were classified as having double function (grey-blue and light grey for upregulated and downregulated genes, respectively). (**E**) Gene Ontology (GO) enrichment analysis of potential miR-21 targets upregulated under stress conditions identified key pathways involved in hepatocarcinogenesis. GO enrichment analysis based on Kyoto Encyclopedia of Genes and Genomes (KEGG) pathways was performed on upregulated genes identified in panels **C**–**E**. Only significant enrichments are represented. The detailed results of these analyses are presented in Figure S3. (**F**) ClueGO+CluePedia (Cytoscape) analysis of upregulated candidates identified numerous functionally grouped network of biological processes (clusters of terms) with putative interactions between them based on their kappa score level (0.45). Node size represents enrichment significance of each cluster (adjusted *p*-value ≤ 0.10). Each cluster of terms is represented by a specific color (detailed in Figure S2). Functionally related terms that overlap in different clusters due to multiple functions of single genes are represented as well. The most representative term of each cluster is highlighted in bold with the respective color attributed to the cluster.

We and others previously reported that miR-21 deficiency in the liver was preventing the development of obesity-associated steatosis, inflammation, and fibrosis, which are important drivers of hepatocarcinogenesis [22,24]. To investigate how miR-21 affects the hepatic microenvironment and potentially carcinogenesis, we performed an RNA sequencing (RNAseq) analysis of hepatic tissues in stress conditions (steatosis induced by 3 weeks of feeding with an obesogenic diet rich in lipids) both in CTRL and LmiR21KO mice. We identified 755 deregulated genes (DEGs, *p*-value < 0.05) between LmiR21KO and CTRL mice, i.e., 355 downregulated and 400 upregulated genes in the liver (Figure 1A). Following GO enrichment analysis, we observed that DEGs in the livers of LmiR21KO mice were frequently involved in metabolic homeostasis, but also in other processes essential for tumor development and/or progression, as well as for regulation of immune responses (e.g., apoptosis, liver regeneration, innate immune response, and response to cytokines) (Figure 1B). To further characterize miR-21-dependent DEGs relevant for hepatocarcinogenesis, we cross-referenced DEGs in LmiR21KO livers with a list of predicted/validated targets of miR-21 obtained from the miRwalk database [42] (Figure 1C). We thereby identified 124 genes of interest (Figure 1C) which were classified as oncogenes (ONCs), tumor suppressors (TSs), or drivers (Ds), i.e., genes shown to be involved in cancer progression when mutated (either ONCs or TSs), based on literature screening and CancerMine database categorization (Figure 1D and Table S3). Strikingly, some ONCs, TSs, and Ds were found to be upregulated and others downregulated in miR-21-deficient hepatic tissues (Table S3) suggesting that miR-21 does not exert a clear oncogenic role in the liver. 

Following GO enrichment analysis with the 124 genes of interest identified in Figure 1C, we observed that proteins encoded by upregulated DEGs were involved in biological pathways and processes potentially promoting carcinogenesis (KEGG pathway gene enrichment analysis) such as cell proliferation and cell survival (Figure 1E,F, Figures S2 and S3). Other cellular processes were also affected by the loss of miR-21, like cell response to stress and cytokines, the cell cycle and wound healing processes typically associated with inflammation/fibrosis development (Figure 1E,F, Figures S2 and S3, Table S4).

These analyses revealed that loss of hepatic miR-21 in stress conditions leads to both up and downregulation of genes (ONCs, TSs, Ds) predicted to exert opposite functions in hepatocarcinogenesis and immune responses.

### 3.2. Whole-Body and Liver-Specific MiR-21 Deficiency Fosters Carcinogen (DEN)-Induced HCC Development in Mice

**Figure 2:**
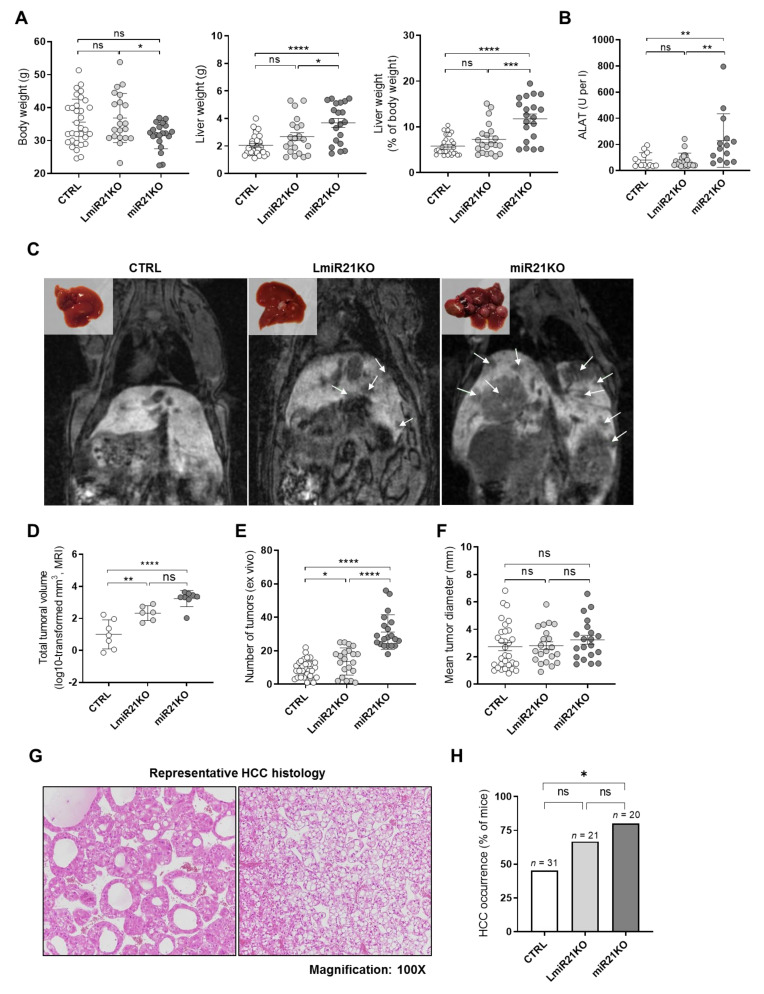
MiR-21 deficiency fosters DEN-induced HCC development. (**A**) Body weight, liver weight, and liver-to-body weight ratio of 11-month-old control (CTRL—miR21fx, *n* = 32), hepatocyte-specific and total miR-21 knockout (LmiR21KO, *n* = 21, and miR21KO *n* = 20, respectively) mice injected with diethylnitrosamine (DEN, 25 mg/kg of body weight) intraperitoneally at day 15 post-birth. (**B**) Plasma levels of alanine-aminotransferase (ALAT) in CTRL (*n* = 27), LmiR21KO (*n* = 21), and miR21KO (*n* = 19) mice. (**C**) Representative pictures of explanted livers post-sacrifice and magnetic resonance imaging (MRI) prior to sacrifice of CTRL, LmiR21KO, and miR21KO mice. Tumoral masses are indicated by white arrows in the MRI scans. (**D**) Volume of the total tumoral mass quantified by MRI (values log10-transformed) in CTRL (*n* = 8), LmiR21KO (*n* = 8), and miR21KO (*n* = 8) mice. (**E**) Number and (**F**) mean diameter of isolated tumors from explanted liver in CTRL (*n* = 32), LmiR21KO (*n* = 21), and miR21KO (*n* = 20) mice. (**G**) Representative histopathological sections (hematoxylin-eosin staining, original magnification ×100) of typical HCC tumors found indiscriminately in HCC in either CTRL, LmiR21KO, or miR21KO mice. Left panel shows HCC in miR21KO with a pseudo-glandular pattern and eosinophilic cells. Right panel shows HCC in miR21KO with trabecular architectures and clear cell changes. (**H**) Percentage of mice in the respective cohorts developing hepatocellular carcinoma (HCC) as determined by histopathological analyses of H&E stained sections (CTRL, *n* = 31; LmiR21KO, *n* = 21, miR21KO, *n* = 20). Data that failed normality tests was submitted to log10-transformation prior to statistical analysis. An outliers test was performed using the ROUT method (Q = 1%). One-way ANOVA with Holm–Sidak’s correction was used for comparison between more than two groups. Chi-square test was used to evaluate the independence of categorical variables (percentage of samples with detected expression). ns, not significant, * *p*-value < 0.05, ** *p*-value < 0.01, *** *p*-value < 0.001, **** *p*-value < 0.0001.

To address the in vivo role of miR-21 in HCC development, we investigated whether whole-body (miR21KO) or hepatocyte-specific (LmiR21KO) *Mir-21a* genetic deletion can affect HCC development induced by DEN. In this mouse model, HCC characterized by Ras mutations (as well as numerous others such as *Braf*, *Egfr*, or *Apc*), develop in almost 100% of animals after 11 months following DEN-injection at the age of 15 days [50,51]. At 11 months post-DEN injection, LmiR21KO tend to have a higher liver weight than their control littermates (miR21fx—CTRL), while miR21KO mice display highly significant hepatomegaly (Figure 2A). The increase in liver weights appears to be mainly driven by a higher tumor burden since no significant differences were observed in the liver weights of CTRL, LmiR21KO, and miR21KO mice in standard breeding conditions without any tumor induction (1.0 ± 0.09, 1.1 ± 0.09, 1.2 ± 0.5 gr for CTL, LmiR21KO, and miR21KO respectively). Serum ALAT measurements indicated elevated liver injury with whole-body miR-21 deficiency (Figure 2B) likely reflecting underlying hepatic stress and injury induced by the concomitant deficiency of miR-21 and the long-term effects of DEN exposure [52]. MRI of mouse livers prior to sacrifice, as well as tumoral nodule counts in explanted livers clearly show that LmiR-21KO mice develop significantly more, but not bigger, tumors than their CTRL littermates (Figure 2C–F). This suggests that tumor initiation is increased when miR-21 is deficient in hepatocytes, but further tumor growth is not affected. The effect on tumor initiation was even more accentuated in mice with a total knockout of miR-21 (i.e., a 2–3-fold increase in both total tumoral mass and tumor numbers; Figure 2C–F) consistent with the observed hepatomegaly. The histopathological analyses of tumoral livers sections from CTRL, LmiR21KO, and miR21KO mice revealed nodular livers at a microscopical level with focal steatosis in all groups. Some of these nodules were hepatocellular adenoma (HCA) while others, significantly less frequent in the CTRL group, were clearly identifiable HCC (Figure 2G). Of note, an increment of >20% and >35% in HCC occurrence was observed in LmiR21KO and miR21KO, respectively, in comparison to CTRL mice, suggesting that malignant transformation of tumoral foci was also increased in the absence of miR-21 (Figure 2H). 

Altogether, these analyses revealed that loss of miR-21 in hepatocytes triggers multiple and pleiotropic effects promoting oncogenesis. Oncogenicity associated with miR-21 deficiency in the liver is further enhanced when miR-21 activity is also abrogated in other relevant cell types contributing to hepatocarcinogenesis, as illustrated by the stronger cancerous phenotype observed in miR21KO total knockout as compared to the liver-specific miR-21 knockout mice.

### 3.3. Hepatocyte-Specific miR-21 Ablation Strongly Exacerbates Inflammation, Fibrosis, and Hepatocarcinogenesis Induced by PTEN Deficiency

**Figure 3:**
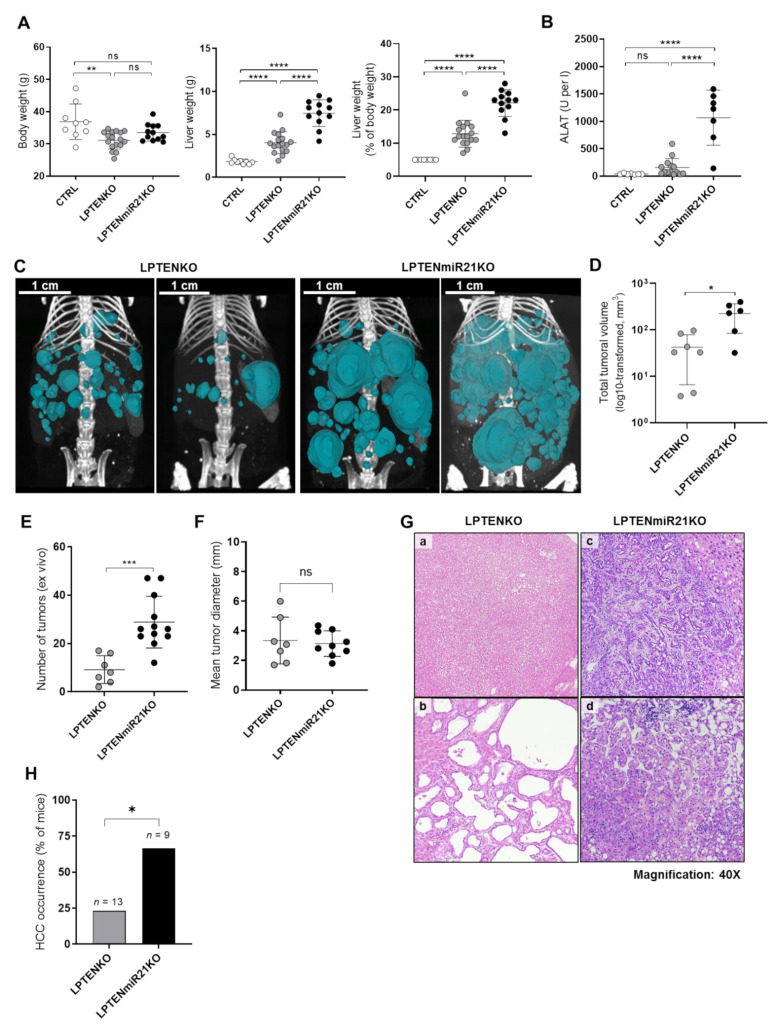
Hepatocyte-specific miR-21 deletion promotes exacerbates hepatocarcinogenesis induced by PTEN deficiency. (**A**) Body weight, liver weight, and liver-to-body weight ratio of control (CTRL—floxed mice not expressing the Alb-Cre recombinase, *n* = 9), hepatocyte-specific PTEN knockout (LPTENKO (*n* = 17), and LPTENmiR21KO (*n* = 12), respectively) sacrificed at 12 months of age. (**B**) Plasma levels of alanine-aminotransferase (ALAT) in CTRL (*n* = 7), LPTENKO (*n* = 14), and LPTENmiR21KO (*n* = 7) mice. Data is represented as mean ± SD. (**C**) Representative three-dimensional reconstructions of computed tomography (CT) scans performed prior to sacrifice. Tumoral masses are represented in blue. (**D**) Volume of the total tumoral mass (values log10 transformed) quantified by CT scanning (LPTENKO, *n* = 7; LPTENmiR21KO, *n* = 6). Data is represented as mean ± SD. (**E**) Number and (**F**) mean diameter of isolated tumors from explanted liver in LPTENKO (*n* = 7 mice) and LPTENmiR21KO (*n* = 12 mice). Data is represented as mean ± SD. Outliers were removed. (**G**) Histopathological characterization (hematoxylin-eosin staining, magnification 40×) of tumoral nodules in the livers of LPTENKO mice (**a**) well-differentiated hepatocellular adenoma (HCA) and (**b**) cholangioma) and LPTENmiR21KO mice (**c**) hepatobiliary tumors of mixed origin and (**d**) HCC). (**H**) Percentage of mice in the respective cohorts developing hepatocellular carcinoma (HCC) as determined by histopathological analyses of H&E stained sections (LPTENKO, *n* = 13; LPTENmiR21KO, *n* = 9). Data that failed normality tests was submitted to log10-transformation prior to statistical analysis. An outliers test was performed using the ROUT method (Q = 1%). Unpaired *t*-test with Welch’s correction was used for comparison between two groups. One-way ANOVA with Holm–Sidak’s correction was used for comparison between more than two groups. Chi-square test was used to evaluate the independence of categorical variables (percentage of samples with detected expression). ns, not significant, * *p*-value < 0.05, ** *p*-value < 0.01, *** *p*-value < 0.001, **** *p*-value < 0.0001.

Mice with a hepatocyte-specific deletion of the tumor suppressor PTEN represent a relevant model of HCC development in a NAFLD context [38,53]. To further assess the tumor-suppressive role of miR-21 in hepatocytes, we generated mice with floxed alleles of the *Pten* and *Mir-21a* genes and expressing a *Cre* recombinase transgene under the control of a hepatocyte-specific albumin promoter (*AlbCre*), thus leading to the deletion of both PTEN and miR-21 specifically in the hepatocytes (LPTENmiR21KO mice). Tumor development was then assessed in LPTENKO mice and in a prospective colony of LPTENmiR21KO mice by CT-scan imaging prior to sacrifice at 12 months of age. At autopsy, we observed that LPTENmiR21KO mice displayed a drastic increase in their liver weight compared to LPTENKO mice, despite having similar body weights (Figure 3A). Liver injury (serum ALAT levels) was also significantly increased in LPTENmiR21KO as compared to LPTENKO mice (Figure 3B). CT-scan analyses revealed that both tumoral numbers and total volume mice were more than two-fold higher in LPTENmiR21KO than in LPTENKO mice (Figure 3C,D and Figure S4A). Increased tumorigenesis in the absence of miR-21 was further confirmed by counting visible tumors in ex-vivo explanted livers (Figure 3E). Nevertheless, no differences were observed in the mean tumor sizes, thus indicating that tumor initiation was fostered, while the growth of established tumoral foci was unaffected by miR-21 deficiency (Figure 3F and Figure S4B) Histopathological analyses of liver sections from LPTENmiR21KO mice revealed a drastic aggravation of the inflammatory and fibrotic phenotype along with abundant immune cells infiltrates in both tumoral and non-tumoral tissues relative to LPTENKO (Figure S5A). In LPTENKO mice, 25% of the tumors could be undoubtedly classified as HCC, with the remaining nodules being mostly well-differentiated HCA or cholangioma (Figure 3G panels a and b respectively, Figure 3H). The latter were observed in LPTENmiR21KO as well. However, a striking 70% of nodules were well-established HCC (Figure 3H) often of poor differentiation (mixed hepatocyte and biliary origin, Figure 3G panels c-d) and accompanied by more severe inflammation/fibrosis (Figure S5B,C). 

These data demonstrate that the loss of miR-21 in Pten-deficient liver strongly promotes the initiation of tumors and their malignant appearance in association with exacerbated inflammation and fibrosis. 

### 3.4. Hepatic miR-21 Suppression Is Associated with Pleiotropic and Context-Dependent Molecular Alterations driving Carcinogenesis

**Figure 4:**
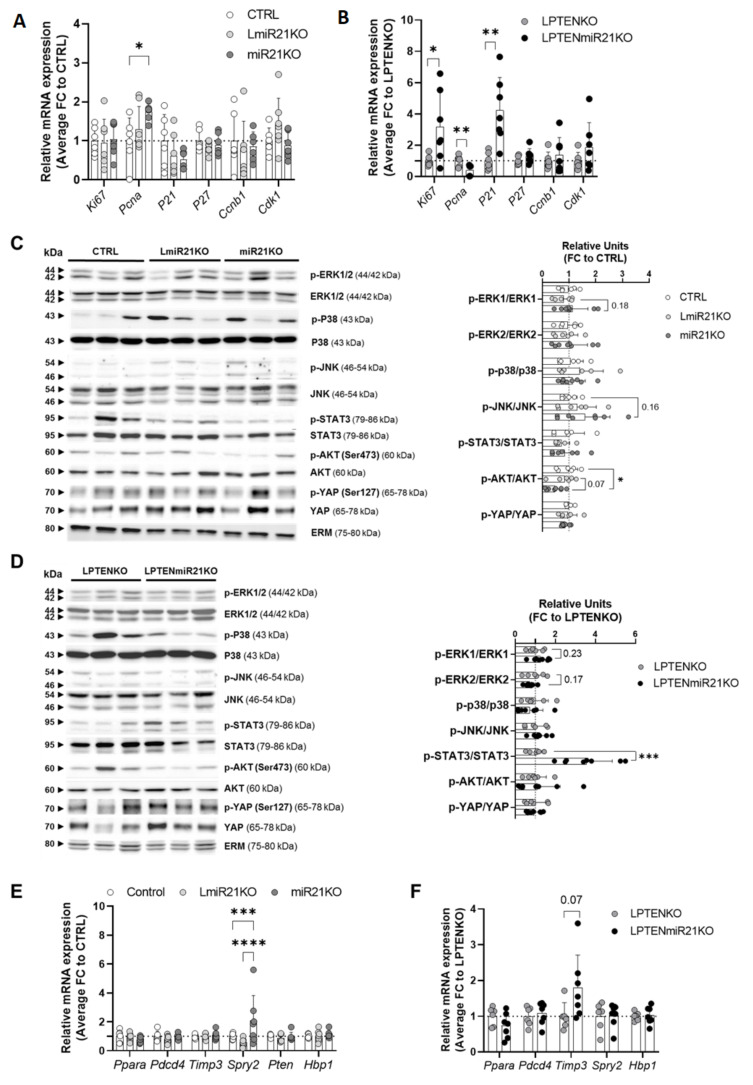
Impact of miR-21 deficiency on cell proliferation, oncogenic signaling pathways, and previously identified miR-21 cancer-related targets. *Ki67, Pcna, p21, p27, Ccnb1, Cdk1* expression in hepatic tissues of (**A**) 11-month-old control (CTRL, *n* = 6), hepatocyte-specific (LmiR21KO, *n* = 7), and total miR-21 (miR21KO *n* = 7) knockout mice injected with DEN and (**B**) 12-month-old hepatocyte-specific PTEN knockout (LPTENKO, *n* = 6) and PTEN/miR-21 knockout (LPTENmiR21KO, *n*=7) mice. (**C**) Representative Western blot (left panel, 3 mice per group) and quantifications (right panel) of phosphorylated over total expression ratio of ERK1, ERK2, p38, JNK, STAT3, AKT, and YAP in hepatic tissues of 11-month-old control, hepatocyte-specific, and total miR-21 knockout mice injected with DEN. Quantifications were performed on Western blot analysis of *n* = 6 CTRL mice, *n* = 6 LmiR21KO, and *n* = 8 miR21KO. (**D**) Representative Western blot (left panel, 3 mice per group) and quantifications (right panel) of phosphorylated over total expression ratio of ERK1, ERK2, p38, JNK, STAT3, AKT, and YAP in hepatic tissues of 12-month-old hepatocyte-specific PTEN knockout and PTEN/miR-21 knockout mice. Quantifications were performed on Western blot analysis of *n*=5 LPTENKO and *n* = 8 LPTENmiR21KO. ERM expression was assessed as a loading control for WB. RT-qPCR analysis of *Ppara, Pdcd4, Timp3, Spry2, Pten*, and *Hbp1* expression in the livers of (**E**) 11-month-old control (CTRL—miR21fx, *n* = 6), hepatocyte-specific, and total miR-21 knockout (LmiR21KO, *n* = 7, and miR21KO *n* =7, respectively) mice injected with DEN and (**F**) in the livers of hepatocyte-specific PTEN knockout (LPTENKO, *n* = 6) and PTEN-miR21 knockout (LPTENmiR21KO, *n* = 7) mice sacrificed at 12 months of age. Data from DEN-injected miR-21 KO mice is represented as mean± SD fold-change (FC) to CTRL mice (miR21fx treated with DEN). Data from LPTENmiR21KO is represented as mean ± SD FC to LPTENKO mice. Data that failed normality tests was submitted to log10-transformation prior to statistical analysis. An outliers test was performed using the ROUT method (Q = 1%). Unpaired *t*-test with Welch’s correction was used for comparison between two groups. One-way ANOVA with Holm–Sidak’s correction was used for comparison between more than two groups. * *p*-value < 0.05, ** *p*-value < 0.01, *** *p*-value < 0.001, **** *p*-value < 0.0001.

Given the wide range of cellular processes which can be affected by the loss of miR-21, based on the hundreds of predicted targets for this microRNA, the increased hepatic carcinogenesis observed in two very different in vivo mouse models of HCC (Figure 2 and Figure 3) was logically expected to be multifactorial. We therefore investigated a panel of molecular alterations in the hepatic tissues of our mouse cohorts that were previously suggested to be impacted by miR-21 and that could potentially prime and foster cancer development. Since miR-21 was previously reported to modulate cell proliferation in vitro [34,54,55,56,57], we thus investigated whether proliferation, cell cycle progression, or apoptosis was affected by miR-21 suppression. While apoptosis, as assessed by different markers (*Bcl2*, *Bclxl*, *Bax*, *Bak*, *Bad*, and *Bid* expression) and caspase 3 cleavage, was not significantly modulated by the absence of miR-21 in both mouse models (Figure S6), cell proliferation and cell cycle progression appear to be promoted, but through slightly different mechanisms in mice exposed to DEN and deficient for PTEN. *Ki67* expression was high in all DEN-treated mice independently of miR-21 expression (Figure 4A and Figure S7) but *Pcna* expression was induced only in miR-21 deficient hepatic tissues of DEN-treated mice (Figure 4A). On the other hand, in PTEN knockout mice we observed a significant induction of *Ki67* expression with a miR-21 deficiency that was accompanied by downregulation of *Pcna* (Figure 4B and Figure S7), consistent with previous studies reporting *Pcna* downregulation in steatotic livers [58,59,60]. 

MiR-21 was also previously reported to regulate pro-oncogenic pathways (MAPK, STAT3, PI3K/AKT, and HiPPO) [28,55,61,62,63], in agreement with our in silico analyses showing that genes upregulated in the absence of miR-21 were involved for example in MAPK, STAT3, and PI3K/AKT signaling (Figure 1F and Figure S3). We thus investigated the expression and activation by phosphorylation of key effectors of these pro-oncogenic pathways by Western blot. Despite a high variability between mice, inherent to these analytical methods, a positive trend for ERK1, p38, and JNK over-activation was observed in DEN-treated LmiR21KO or miR21KO mice as compared to CTRL mice (Figure 4C). In contrast, deletion of miR-21 in LPTENKO mice was associated with a strong and significant activation of STAT3 and a trend for ERK1, JNK, and AKT over-activation (Figure 4D). Of interest, while in hepatic tissues of both HCC models the ratio of phosphorylated Yap over the total was unchanged, expression levels of Yap were increased (average increase of 46% in LmiR21KO, 37% in miR21KO and of 42% in LPTENmiR21KO) in the absence of miR-21 consistent with an inactivation of the HiPPO pathway. 

Although statistical significance could not always be reached in our analyses of one-year-old mouse tissues (potentially due to the versatility of Western blots analyses and the low number of available mouse tissues), it is likely that even a weak over-activation or inhibition of these pathways over the one year of time required to develop tumors in our models can significantly impact the tumor burden and progression.

Finally, we also investigated the expression of other cancer-related miR-21 targets, which were previously shown to be regulated at the mRNA level by miR-21 and reported to act mostly as tumor suppressors in vitro. Among those, i.e., *Ppara*, *Pdcd4*, *Timp3*, *Spry2*, *Pten*, and *Hbp1* [23,64,65,66], we could observe only a significant increase of *Spry2* and a positive trend for *Timp3* expression in hepatic tissue of miR21KO and LPTENmiR21KO mice, respectively (Figure 4E,F and Figure S8A,B). Our data indicate that although miR-21 overexpression may target these tumor suppressors, as previously demonstrated in vitro, suppression of miR-21 in vivo and within the liver microenvironment is not associated with an increased expression of these tumor-suppressive factors and restrained tumorigenesis.

### 3.5. Increased Expression of Oncogenes and Deregulation of Immunity-Related Factors Are Associated with the Loss of miR-21 in the Liver

**Figure 5:**
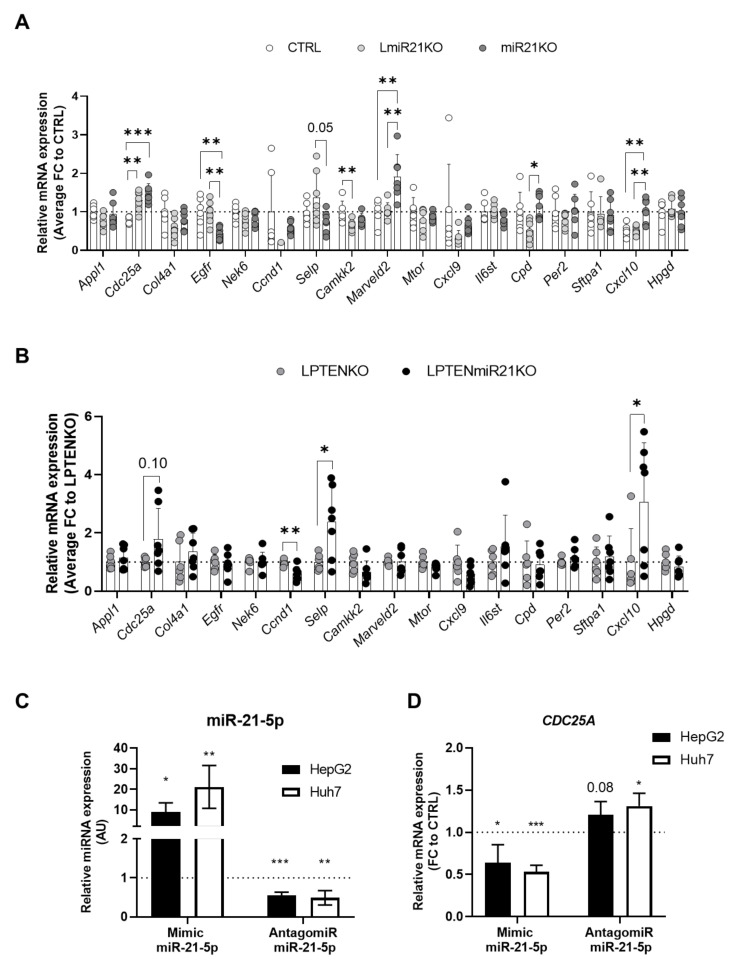
miR-21 deficiency in vivo is associated with *Cdc25a* overexpression and deregulated expression of immunity-related factors in the liver. RT-qPCR analysis of *Appl1, Cdc25a, Col4a1, Egfr, Nek6, Ccnd1, Selp, Camkk2, Marveld2, Mtor, Cxcl9, Il6st, Cpd, Per2, Sftpa1, Cxcl10,* and *Hpgd* expression in hepatic tissues of (**A**) 11-month-old control (CTRL, *n* = 6), hepatocyte-specific (LmiR21KO, *n* = 7), and total miR-21 (miR21KO *n* = 7) knockout mice injected with DEN and (**B**) 12-month-old hepatocyte-specific PTEN knockout (LPTENKO, *n* = 6) and PTEN/miR-21 knockout (LPTENmiR21KO, *n* = 7) mice. Data from DEN-injected miR-21 KO mice is represented as mean ± SD fold-change (FC) to DEN-treated CTRL mice. Data from LPTENmiR21KO is represented as mean ± SD FC to LPTENKO mice. (**C**) miR-21-5p expression and (**D**) *CDC25A* expression in HepG2 and Huh7 hepatic cancer cell lines, transfected for 48 h under basal culturing conditions with either miR-21-5p mimicking oligonucleotides (mimic) or miR-21-5p inhibiting oligonucleotides (antagomir). *CDC25A* expression is represented as mean ± SD FC to Control Mimic or Control Antagomir. Data that failed normality tests was submitted to log10-transformation prior to statistical analysis. An outliers test was performed using the ROUT method (Q=1%). Unpaired *t*-test with Welch’s correction was used for comparison between two groups. One-way ANOVA with Holm–Sidak’s correction was used for comparison between more than two groups. * *p*-value < 0.05, ** *p*-value < 0.01, *** *p*-value < 0.001.

In addition to the potential miR-21 targets analyzed in the previous section, we further assessed the expression of candidate ONCs, TSs, and D uncovered in our transcriptomic analyses as miR-21 targets potentially modulated in vivo (Figure 1 and Figure S1, Table S3). Consistent with a pro-tumorigenic environment developing in miR-21-deficient hepatic tissues, we found that mRNA expression of the oncogenes *Cdc25a* and *Marveld2* and of the driver *Cpd* was significantly increased in DEN-treated mice (Figure 5A), while only *Cdc25a* expression was increased in PTEN-deficient mice when miR-21 was absent. CDC25A is of particular interest since miR-21 was already previously shown to restrain carcinogenesis in colon and breast cancer, in part by repressing the expression of CDC25A [56,57]. Since miR-21 targeting of *Cdc25a* was never demonstrated in the liver, we further confirmed in vitro, using two different hepatic cell lines (i.e., HepG2 and Huh7 cells), that synthetic nucleotides either mimicking or inhibiting miR-21 were able to respectively significantly decrease or increase *Cdc25a* expression in hepatocytes (Figure 5C,D). Finally, besides *Cdc25a*, and consistent with the detected over-activation of STAT3 in LPTENmiR21KO mice (Figure 4D), deregulations of genes involved in inflammation/immune responses such as the selenoprotein *Selp* and *Cxcl10* cytokine, were also observed in the livers of both HCC mouse models (Figure 5A,B) further suggesting deregulated immune responses with cancer development when miR-21 is suppressed. 

### 3.6. MiR-21 Deletion Differentially Affects Inflammatory and Immune Responses Induced by Acute DEN Exposure and PTEN Deficiency in the Liver

Based on our in silico analyses of inflammatory/immune pathways performed in Figure 1 and Figures S2 and S3, activation of the STAT3 pathways in miR-21-deficient LPTENKO (Figure 4D), immune cell infiltrations observed in histological sections (Figure S5), and deregulated cytokines expression in miR-21-deficient liver samples (Figure 5A,B), we hypothesized that inflammatory responses and immune cells recruitment in miR-21 deficient liver tissues could be impaired. Supporting this hypothesis, miR-21 was previously reported to modulate immune responses [27,67,68] and miR-21 deficiency appears to affect mostly tumor initiation, but not tumor growth. We therefore examined how miR-21 loss affects inflammatory and immune responses in pre-tumoral hepatic tissues.

Immune cell infiltration and activation in response to an acute exposure of DEN in CTRL, LmiR21KO, and miR21KO mice were examined 48 h post-DEN-injection in adult mice, as suggested by previous studies [40]. As expected, DEN induced acute liver injury (serum ALAT levels, Figure S9A), but surprisingly less pronounced in miR21KO mice (46% less, *p* = 0.062) as compared to CTRL and LmiR21KO mice (Figure S9A), suggesting less DEN-induced acute cell injury in miR21 total knockout mice. 

**Figure 6:**
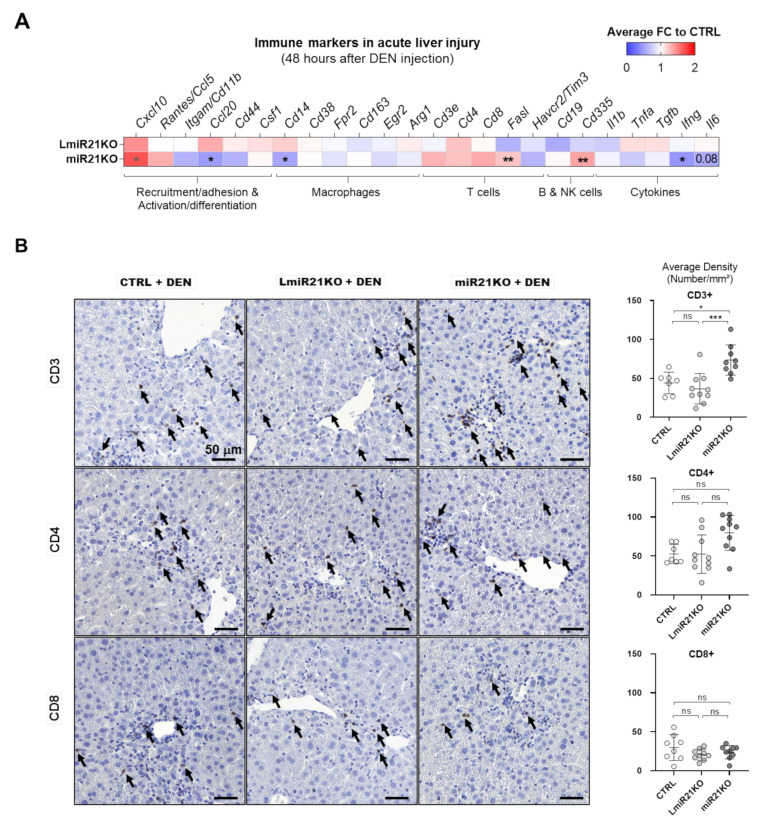
MiR-21 deficiency alters DEN-induced acute inflammatory responses and immune cells recruitment in the liver prior to tumor initiation. (**A**) Heatmap representation of relative mRNA expression of inflammatory markers involved in immune cell recruitment, adhesion, activation, and differentiation; of macrophages, T cells, B cells and NK cells; and of pro- and anti-inflammatory cytokines in non-tumoral hepatic tissues from 2-month-old mice 48 h post-DEN injection (100 mg/kg of body weight; CTRL, *n* = 9 mice, LmiR21KO, *n*=10 mice; miR21KO mice, *n* = 10 mice). Data are represented by mean fold-change (FC) to CTRL (miR21fx treated with DEN). (**B**) Representative immunohistochemical staining of CD3, CD4 and CD8 in liver sections (left) and quantification of positive cells (number of positive cells per mm^2^, right) in DEN-injected mice (CTRL, *n* = 8; LmiR21KO, *n* = 5–10, miR21KO, *n* = 7–9). Data is represented as mean ± SD. Data that failed normality tests was submitted to log10-transformation prior to statistical analysis. An outliers test was performed using the ROUT method (Q = 1%). One-way ANOVA with Holm–Sidak’s correction was used for comparison between more than two groups with one variable. * *p*-value <0.05, ** *p*-value < 0.01, *** *p*-value < 0.001.

Analysis of several markers of immune cell recruitment, adhesion, and activation in CTRL liver two days post-DEN exposure reflected the development of the expected inflammatory response (increased expression of *Cxcl10*, *Rantes*/*Ccl5*, *Itgam*/*Cd11b*, *Ccl20*, *Cd44*, *Csf1*; Figure S9B). Mostly similar expression patterns were observed for LmiR21KO liver, however, in miR21KO some of the immune markers (i.e., *Itgam*/*Cd11b*, *Ccl20*, *Cd44*) were downregulated, suggesting that DEN-induced acute inflammatory processes were attenuated in miR-21 total knockout mice (Figure 6A). However, the expression of T-cell markers (*Cd3e*, *Cd4*, and *Cd8*) tended to be increased in miR21KO relative to LmiR21KO liver, an observation which was further supported by immunohistochemical quantifications of CD3, CD4, but not CD8, on histological liver sections 48 h post-DEN exposure (Figure 6B). Finally, although we could not observe statistically significant differences in the expression of inflammatory cytokines (*Il1b*, *Tnfa*, *Tgfb*, *Ifng*, *Il6*) or markers for pro-inflammatory M1 (*Cd38*, *Fpr2*) or anti-inflammatory M2 (*Egr2*, *Cd163*, *Arg1*) macrophages, the *Cd14* general macrophage marker was decreased in miR21KO relative to LmiR21KO liver, while markers for NK cells (*Cd335*, *Klrb1c*) followed an opposite trend (Figure 6A and Figure S10A,B). 

**Figure 7:**
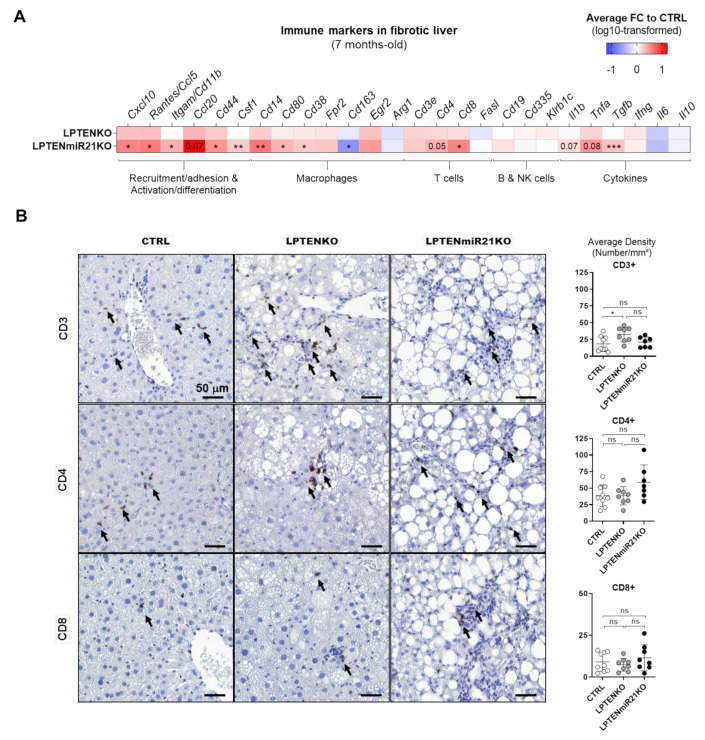
Loss of miR-21 in PTEN-deficient hepatocytes prevents macrophages and increases T-cell recruitment in pre-tumoral hepatic tissues. (**A**) Heatmap representation of relative mRNA expression of inflammatory markers involved in immune cell recruitment, adhesion, activation, and differentiation; of macrophages, T cells, B cells and NK cells; and of pro- and anti-inflammatory cytokines in pre-tumoral hepatic tissues of 7-month-old mice (CTRL, *n* = 9; LPTENKO, *n* = 8; LPTENmiR21KO, *n* = 12). Data were submitted to log10-transformation of the fold-change (FC) to CTRL and is represented as means. (**B**) Representative immunohistochemical staining of CD3, CD4, and CD8 in liver sections (left) and quantification of positive cells (number of positive cells per mm^2^, right) in CTRL (*n* = 9), LPTENKO (*n* = 8), and LPTENmiR21KO (*n* = 7–8) mice. Data is represented as mean ± SD. Data that failed normality tests was submitted to log10-transformation prior to statistical analysis. An outliers test was performed using the ROUT method (Q = 1%). Unpaired *t*-test with Welch’s correction was used for comparison between two groups. One-way ANOVA with Holm–Sidak’s correction was used for comparison between more than two groups. * *p*-value < 0.05, ** *p*-value < 0.01, *** *p*-value < 0.001.

Similar analyses were performed for 7-month-old LPTENKO and LPTENmiR21KO mice (just before tumors could be detected by CT-scan imaging) to evaluate the immune microenvironment of developing tumors. Consistent with the massive immune infiltration, fibrosis, and with over-activation of STAT3 signaling observed in 1-year-old LPTENmiR21KO mice (Figure 4D and Figure S5A–C), non-tumoral liver tissues from LPTENmiR21KO mice displayed a marked recruitment of immune cells as compared to LPTENKO mice. Markers of immune cell recruitment (*Cxcl10*, *Rantes*/*Ccl5*, *Ccl20*), adhesion (*Itgam*/*Cd11b*, *Cd44*), and macrophage recruitment/differentiation (*Ccl1*, *Cd14*, *Csf1*) were all significantly upregulated in LPTENmiR21KO relative to LPTENKO liver (Figure 7A and Figure S11A,B). Strikingly, LPTENmiR21KO liver displayed a significant upregulation of M1 markers (*Cd80* and *Cd38*) at the expense of M2 markers (*Cd163* and *Arg1*), consistent with a pro-inflammatory environment and the observed enhanced expression of pro-inflammatory cytokines (*Il1b* and *Tnfa*) (Figure 7A).

RT-qPCR analysis was consistent with higher T-cell infiltration in hepatic tissues of LPTENmiR21KO mice (*Cd3e*, *Cd4,* and *Cd8*), which was further supported by a trend to increased CD4 and CD8 on immunohistochemistry (Figure 7B). Of note, *Foxp3*, a marker for regulatory T cells (T reg), was also significantly increased in the absence of miR-21, pointing to potentially compromised anti-tumoral T-cell responses in LPTENmiR-21KO mice (Figure S11C,D). 

Altogether, these data suggest that miR-21 deficiency in hepatocytes might foster tumorigenesis in the liver by compromising inflammatory and immune responses, which could be further exacerbated by miR-21 deficiency in non-hepatocyte cells. Likely, the underlying mechanisms will be pleiotropic and complex, with dependency on the cellular context, the type of insults, and the stage of the disease.

## 4. Discussion

**Figure 8:**
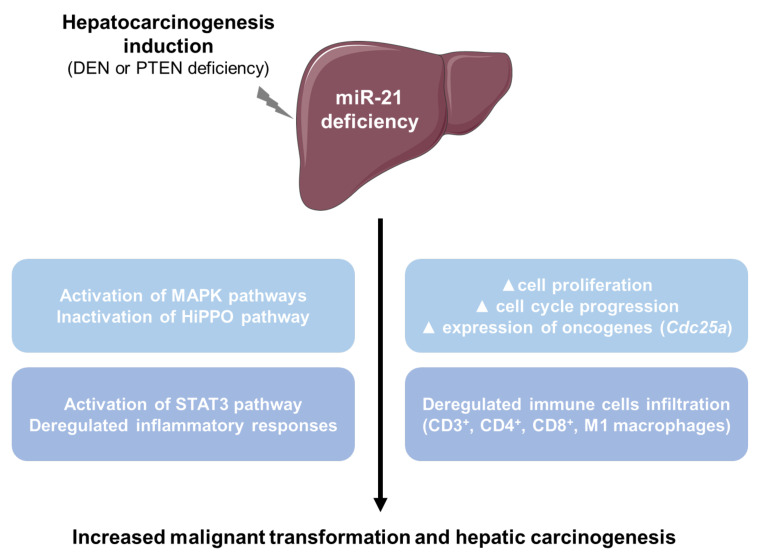
miR-21 deficiency promotes hepatocarcinogenesis in two distinct HCC animal models through pleiotropic mechanisms. These mechanisms involve subtle alterations of pro-oncogenic (MAPK, HiPPO pathways) and pro-inflammatory signaling pathways (STAT3 pathways), increased cell proliferation and cell cycle progression, increased expression of oncogenes (e.g., *Cdc25a*), and deregulated immune cells’ infiltration.

In this study, we provide solid in vivo evidence that miR-21 deficiency unexpectedly favors liver cancer development. The frequent upregulation of miR-21 in primary tumors and blood of individuals diagnosed with HCC, which also correlated with disease progression, has led to the extrapolation that miR-21 has an oncogenic function in the liver, as previously described for other cancers [20,25,29,69,70,71,72]. Here we show that miR-21 expression in hepatocytes can have, on the contrary, a tumor-suppressive function regardless of HCC etiology, since in vivo silencing of miR-21 specifically in hepatocytes fosters tumor occurrence and malignancy in both genetic (LPTENKO mice) or carcinogen-induced (by DEN) murine models of HCC through pleiotropic mechanisms (Figure 8).

A tumor suppressive function of miR-21 in liver cancer may appear paradoxical given the suggested oncogenic role for miR-21 in other cancers and in cultured hepatic cells [26,32]. However, in addition to the data presented here, several other studies have contradicted this dogmatic view. Indeed, Schipper et al. recently reported in pancreatic cancer that miR-21 deficiency enhanced tumorigenesis through processes associated with increased mucinous cystic neoplastic lesions, an absence of tumor-restraining myofibroblasts, and a significant infiltration of immune cells [73]. In colorectal cancer cell lines, the ablation of the *miR-21* gene was shown to induce an upregulation of *CDC25A* expression, which triggered cell cycle progression (G1-S and G2-M) and an increased proliferation [57]. Similar findings were further reported in breast cancer cells, in which exposure to a phytochemical (3,3’-Diindolylmethane) led to miR-21 upregulation. In this case, miR-21 was also shown to target *CDC25A* thereby promoting cell cycle arrest and a decreased proliferation in vitro, as well as in a xenograft model [56]. To our knowledge, only two studies evaluated, with controversial results, the outcomes of miR-21 inhibition in HCC development using in vivo models instead of xenografts. First, miR-21 inhibition mediated by synthetic inhibitory nucleotides (antagomiRs) was reported by Zhang and co-authors to prevent liver carcinogenesis in hepatocyte-specific *Pten* knockout mice. Although the miR-21 network of targets involved in this protective effect could not be identified, administration of these antagomiRs was associated with an increased apoptosis of CD24+ progenitor cells [36]. We do not have a clear explanation for this phenotype opposite to our data, but discrepancies between in vivo studies using synthetic miR-21 inhibitors and genetic silencing of the *Mir-21a* gene have already been reported for cardiac fibrosis [74,75] possibly due to off-target inhibition of mRNA species harboring seed sequences close to those of miR-21 [76]. In addition, antagomiRs against miR-21 are probably inhibiting miR-21 in several liver cell types and maybe also extrahepatic cells, unlike the hepatocyte-specific genetic deletion in our LmiR21KO and LPTENmiR21KO mice. In this regard, nucleotide-mediated inhibition of miR-21 in biliary and liver inflammatory cells was shown to prevent the development of hepatic inflammation/fibrosis [21], two conditions promoting HCC development [2]. The anti-fibrotic effects of miR-21 synthetic inhibitors might therefore contribute to their anti-carcinogenic action [36]. Finally, genetic deletion of the *MiR-21a* gene in our miR21KO, LmiR21KO, and LPTENmiR21KO mice also prevents expression of the passenger strand miR-21*, which is not inhibited by antagomiRs against the miR-21-5p. Interestingly, miR-21* (or miR-21-3p) is now reported as a functionally relevant passenger strand, as shown in human HepG2 hepatoblastoma cells where it inhibited growth and triggered apoptosis [77], but promoted, in another study, migration and invasion of the same cells [63]. MiR-21* was also reported to play a significant role in other processes such as cardiac and renal fibrosis [78,79], hypertension [80], or inflammation [81,82,83]. We cannot exclude that the in vivo genetic deletion of miR-21* in hepatocytes also contributes to the exacerbated hepatocarcinogenesis seen in miR21KO, LmiR21KO, and LPTENmiR21KO mice. Further studies are, however, required to assess this hypothesis, in particular given the emergence of therapeutic strategies aiming at preventing the maturation of the primary transcript of miR-21 (pri-miR-21) and its stem-loop precursor (miR-21) [84,85,86]. In a second study, Caviglia et al. reported that the genetic deletion of the miR-21 precursor, or miR-21-5p inhibition by antagomiRs, did not affect tumor development in a model of chemically induced hepatocarcinogenesis elicited by DEN and multiple CCl4 injections in mice [37]. In the same study, authors found that miR-21 was not relevant for HSC activation and liver fibrosis, in contrast to what was reported by others [24,35,36]. Here again, we believe that the impact of miR-21 in inflammation and cancer is strongly context-dependent and it is possible, for example, that repeated injections of CCl4, in addition to DEN (to generate more rapidly hepatic cancers in mice) overcome and mask a normal regulation of these pathological processes by miR-21.

Actually, most of the studies supporting an oncogenic role for miR-21 in hepatic carcinogenesis were performed using cultured hepatoma cell lines and/or their xenografts in mice [28,30,31,32]. Cultured hepatic cancer cells are already transformed, with numerous heterogenous mutations and altered karyotypes, they present considerable metabolic reprogramming and are deprived of interactions with other liver non-parenchymal and immune cells [87,88,89]. MiR-21 activity and its targeted transcripts are highly dependent of the cellular context and microenvironment. Therefore, studies with in vitro cultured cells only poorly recapitulate the hepatic carcinogenesis in vivo, which is highly dependent on other liver cell types and the tumor microenvironment (e.g., inflammation) and may thus lead to inappropriate or false interpretations of the real function of this miRNA in vivo. We previously reported such discrepant results for another important post-transcriptional regulator, which acts similarly to a miRNA, i.e., the RNA-binding protein tristetraprolin (TTP). In vivo studies using TTP knockout mice indicated an oncogenic function for this protein in HCC development, whereas TTP appeared to have a tumor-suppressive role in hepatic cultured cancer cells [90].

Changes in the transcriptome of specific cells, following cell transformation, or upon other pathological stimuli, might therefore strongly influence the set of genes regulated by miR-21, as well as its activity/expression [27,68,71,91]. The importance of the cellular context and extracellular environment for miR-21 functions is further illustrated by the observation that miR-21 overexpression promoted hepatocyte proliferation after partial hepatectomy, and fibrosis by activating HSCs [91]. However, miR-21 overexpression after partial hepatectomy upon ethanol-induced liver injury triggered, on the contrary, an anti-proliferative effect and blocked HSC activation [91]. A similar dual-faceted role for miR-21 was also observed for immune cell functions [92]. Finally, a myriad of other regulatory mechanisms are likely modulating the role and function of miR-21 in the liver, depending on the pathophysiological context. These include in particular competition for the same mRNA targets with other endogenous molecules (RNA-binding proteins, miRNAs, competing-endogenous RNAs, long non-coding RNAs), as well as, for example, editing of miRNA seed sequences or target’s MRE [16,72,93,94]. Such miR-21 competitor molecules are highly regulated depending on the experimental settings, genetic background of the mice, and even on miR-21 expression [4,26]. Feedback regulatory mechanisms of miR-21 expression and activity further complicate the role of this miRNA particularly in multifactorial pathological processes such as inflammation and cancer [27].

The context-dependent functions of miR-21 are further supported by the differences that we observed between our two mouse models of HCC. Although the final outcome of miR-21 deficiency, i.e., an increased hepatic carcinogenesis, were the same in DEN-treated mice and mice bearing a hepatic deletion of PTEN, deregulated mechanisms, which likely contribute to the increased carcinogenesis, were not always similar in both HCC mouse models. We indeed have observed significant differences for example in the expression of specific oncogenes, activation of inflammatory pathways, immune cell recruitment, or upregulation of proliferative markers. One puzzling aspect in particular concerns the different regulation of the *Ki67* and *Pcna* proliferative markers. Actually, while *Ki67* is a general proliferation marker mostly associated with cell mitosis, *Pcna* is a specific marker of the G1 to S phase transition reflecting cell cycle progression [95], but also a marker of activated DNA repair mechanisms [95]. Since miR21KO mice appear to have significant DEN-induced hepatocyte injury (i.e., increased ALAT levels), it is likely that oxidative stress and DNA damage are significantly increased, thus promoting a stronger activation of DNA repair mechanisms, consistent with an increased *Pcna* expression in these mice (Figure 4A). The observed tendency in JNK activation (which is a stress-activated MAPK and sensor of DNA damage [96]) observed in Figure 4C further supports this interpretation. In contrast, in LPTENmiR21KO mice, while *Ki67* is increased as expected, we have observed a downregulation of *Pcna* expression. It is possible that in this case, *Pcna* downregulation might result from the aggravation of steatosis, inflammation, and fibrosis in miR-21-deficient PTEN knockout liver tissues. Indeed, a strong downregulation of hepatic *Pcna* was previously reported in steatotic livers of obese and steatotic *ob/ob* mice [58] or mice fed an obesogenic diet [59]. As well, PCNA downregulation was observed in the liver with steatohepatitis and fibrosis in HCV-infected patients [60]. These observations, in both patients and animal models, suggest that *Pcna* expression in hepatocytes is strongly affected upon severe steatosis, inflammation, and fibrosis. Thus, although the complex mechanisms of *Pcna* regulation still remain unclear, our data appear at least to be consistent with previous observations. 

Based on transcriptomic studies, miR-21 expression was found frequently, but not always, to be upregulated in human cohorts of HCC patients and correlations with specific features like tumor size or cancer stage were established in some cases [70,97,98]. These data are, however, challenged by other studies suggesting that miR-21 levels do not correlate with patient survival [98] or that miR-21 itself does not have sufficient specificity to be considered as a good diagnostic marker of HCC [99,100]. Our analyses of publicly available datasets of the transcriptome of HCC tissues in patients further have indicated that miR-21 expression in HCC is quite heterogeneous and not always upregulated depending on the cohort of patients (Figure S12). Whether the observed miR-21 upregulation in cancer means that this miRNA is oncogenic, or on the contrary, a defense response of the organism to counteract tumorigenesis, is further unclear and remains to be investigated. As well, miR-21 expression in specific tissues and secretion in the blood does not always correlate, as we previously demonstrated for patients infected with HCV [101], thus complexifying the interpretation of the functional role of miR-21 in the tissue of interest. Finally, whether upregulated miR-21 is really active in normal or transformed hepatic cells is still debated. Indeed, miR-21 was previously reported to remain inactive in hepatocytes, despite being well expressed, and to be activated only upon particular stress [101,102]. Such features regarding miR-21 activity were never investigated in hepatic cancers. As well, in thyroid cancer, although an oncogenic role for miR-21 was suggested [103], others reported that miR-21 was not located in the active counterpart of the RNA-induced silencing complex (RISC) [104,105], indicating that despite its overexpression, miR-21 is inactive in this cancer. In MCF-7 cells also, miR-21 was shown to be sequestered by the RNA-binding protein HuR, thereby preventing mRNA decay of *PDCD4*, a classical target of miR-21 [93]. Of note, HuR is strongly upregulated in human HCC, where it correlates with a poor prognosis [106]. It is now also clear that in specific pathological situations mRNAs targeting by miR-21 is likely a matter of a delicate stochiometric equilibrium depending on the amount of miR-21 available, the amount of mRNA target molecules and their competition to bind miR-21, as well as the titration of free miR-21 by irrelevant targets or pseudogenes [107]. Together, these studies indicate that upregulation of miR-21 expression does not always reflect an increased activity. Consistent with such regulatory mechanisms, we previously demonstrated that, although miR-21 expression was not increased in HCV-infected hepatocytes, its activity was strongly promoted by this virus [101].

Our in vivo data have clearly demonstrated that miR-21 deficiency over time fosters hepatic tumorigenesis, but delineating the molecular mechanisms behind this tumor-suppressive function of miR-21 remains highly challenging. Analyses of our transcriptomic data have allowed us to identify several new potential targets of miR-21 in the liver with opposite functions in cancer, based on the literature. However, these analyses do not enable to precisely identify which of these deregulated targets are the most relevant for the observed phenotype. Based on the numerous predicted and/or validated targets for miR-21 and the well-established feature of microRNAs, which exert only a fine-tuning of the expression of their targets, it is therefore highly probable that miR-21 deficiency slightly affects a variety of cellular processes that synergistically enhance carcinogenesis. This conclusion is supported by the relatively weak effects of miR-21 deficiency that we have observed on the activation of oncogenic pathways or the expression of specific genes. Our data has highlighted, however, the over-activation of the STAT3 pathway, in the absence of miR-21 in a context of fatty liver disease induced by the loss of *Pten.* As well, miR-21 deficiency has also triggered an inactivation of the HiPPO pathway as reflected by the increased total expression of Yap in hepatic tissues. In this regard, Yap was shown to be targeted by miR-21 [36,37] and inactivation of the HiPPO pathway in the liver is oncogenic [38] in agreement with the observed increased tumorigenesis in miR-21 KO mice. Finally, *Cdc25a* was the only miR-21 oncogenic target upregulated in both hepatocarcinogenic mouse models investigated in our study. CDC25A is of particular interest since it is a well-characterized oncogene promoting cell cycle progression in cancer cells [108,109,110] and because miR-21 was already previously shown to restrain carcinogenesis in colon and breast cancer, in part by repressing the expression of this specific oncogene [56,57]. *Cdc25a* may thus represent a major driver fostering HCC development in mice. Of note, the expression of previously well-established in vitro targets of miR-21 having tumor-suppressive activity, i.e., *Ppara* [21], *Pdcd4* [64], *Timp3* [64], *Spry2* [62,65], *Pten* [66], and *Hbp1* [23], was unchanged with miR-21 deficiency in vivo in mice again highlighting a cell/organ context-dependent action of miR-21. Whether miR-21 upregulation in vivo is the leading cause of the downregulation of these tumor suppressors in cancer remains to be firmly established, but based on our results, miR-21 inhibition is unlikely to affect the expression of these tumor suppressors beyond their physiological levels in order to counteract cancer development. 

Further complexifying the interpretation of these data is also the recently highlighted conflicting roles of canonical oncogenes reported in liver carcinogenesis. Indeed, other well-characterized oncogenes were shown to dampen hepatic carcinogenesis in a cell type-, cancer stage-, and environment-dependent manner [11]. For example, shutting down the NF-κB pathway prevented HCC development in *Mdr2*-knockout mice [111], but fostered DEN-induced carcinogenesis [112]. Knockout of JNK kinases [113,114], STAT3 [115,116], and β-catenin [117,118] led to either increased or decreased DEN-induced HCC development in mouse depending on the cell specificity of the knockout. Such unexpected data were not limited to the DEN-induced model of HCC, since genetic deletion in hepatocytes of the *Akt1* oncogene in a whole-body *Akt2* knockout context also caused spontaneous HCC development [119]. The loss of these pro-survival factors was associated with a high hepatocyte sensitivity to inflammation-driven cell death, thus potentially explaining the observed tumor-suppressive functions of these canonical oncogenes [11]. 

Together, these data do not point to a specific mechanism under the control of miR-21, which might be majorly responsible of the observed cancer phenotype, but rather to a myriad of slightly deregulated cellular processes, normally fine-tuned by miR-21, and which together contribute to the tumor initiation/progression over time. The hundreds of targets predicted for miR-21 and the numerous ones suggested by experimental approaches are consistent with such a pleiotropic action of this miRNA. 

Consistent with the suggested miR-21 functions in tissue inflammation and fibrosis, miR-21 upregulation was also observed in hepatic stellate cells (HSCs) and in inflammatory cells upon various stimuli [21]. Actually, miR-21 was shown to be involved in HSC activation, T-cell activation and proliferation, macrophage polarization, and pro/anti-inflammatory cytokine expression [35,67,68,120]. As we did not expect increased immune infiltration, fibrosis, and tumorigenesis in the absence of miR-21, our experimental settings enabled us to only assess the expression of markers for immune cell infiltration and activation in ex-vivo explanted whole liver tissue. However, we have observed significant differences in the profiles of immune cell infiltrates within hepatic tissue of total miR-21 knockout mice (miR21KO) as compared to LmiR21KO mice, which likely contribute to the exacerbated cancerous phenotype of miR21KO mice. In particular, markers for monocytes/macrophages were decreased, while markers for NK and T cells, in particular CD4+ T cells, were upregulated, consistent with the strong increase of *Cxcl10* expression. Besides its suspected role in macrophage polarization, miR-21 was also reported to be required for the anti-tumoral activity of tumor-associated macrophages (TAMs) [121]. It is currently unclear whether miR-21 is involved in the efficient recruitment of macrophages in damaged tissue, but the miR-21 deficiency in TAMs was shown to promote a pro-inflammatory microenvironment and recruitment of T cells, consistent with the increased infiltration of CD4+/CD8+ T cells that we have observed [121]. However, miR-21 was further shown to be required to fully activate T cells and to convey their cytotoxic/anti-tumoral functions [68]. In this regard, a study analyzing cancer progression of allograft hepatoma tumors in miR-21-null mice showed that CD4+ and CD8+ T cell responses were weaker in these mice, allowing for a better progression of the allografts, whereas introducing WT CD4+ and CD8+ T cells reversed the phenotype [68]. Since in our miR21KO model, T cells also lack miR-21 expression, it is likely that they are not fully functional, despite a better tissue recruitment, translating in tumor evasion from immune surveillance and response. A similar immune phenotype was observed in a model of pancreatic cancer in mice with a whole-body miR-21 knockout [73]. 

Immune cell infiltration in pre-tumoral tissues was even more evident in mice lacking both PTEN and miR-21 in hepatocytes (LPTENmiR21KO) as compared to the single knockout of PTEN (LPTENKO). In LPTENmiR21KO pre-tumoral hepatic tissues, not only T cells, but also macrophages with an M1 pro-inflammatory polarization profile were abundantly recruited to pre-tumoral hepatic tissues. This severe phenotype is likely the result of a synergic effect of both miR-21 and PTEN deletion in hepatocytes. Previous studies indeed highlighted an increased macrophage infiltration in prostate cancer associated with PTEN loss, but whether the macrophages were polarized toward the M1 or M2 phenotype was not assessed [122]. Our study suggests that miR-21 deficiency may contribute to this M1 polarization, but extensive studies are now required to delineate the mechanistic details. Of note, one interesting track to follow is provided by studies reporting that M1/M2 polarization can be modulated by miR-21-containing exosomes secreted by damaged cells [27,123,124] and that macrophage uptake of mesenchymal stem cell-derived miR-21 attenuated inflammation due to M2 macrophages polarization in a mouse model of sepsis [123]. Finally, we have also observed an increase in recruitment of T cells (upregulation of *Cd3*/*Cd4*/*Cd8* markers) in pre-tumoral tissues of LPTENmiR21KO, but these were associated with an increased expression of *Foxp3*, a marker of regulatory T cells, which are reported to counteract the anti-tumoral cytotoxic effect of CD8+ T cells [125]. Whether these T regs contribute to the exacerbated carcinogenesis observed in LPTENmiR21KO remains to be investigated in depth, but in this regard, infiltration of T regs and exhausted CD8+ T cells in peri-tumoral and tumoral tissues of individuals diagnosed with HCC was shown to impair anti-tumoral responses, therefore promoting disease progression and worsening the disease prognosis [126]. 

Currently there are no in vivo mouse models reflecting accurately the molecular features and heterogeneity of HCC with a specific etiology, or of a defined histological subtype in humans. Each animal model exhibits advantages/disadvantages that need to be considered with care and cautiousness when interpreting experimental results and extrapolating them to the human disease [127,128,129]. We have therefore tried to overcome part of these limitations by analyzing two different mouse models commonly used in the field, but recapitulating distinct features of human HCC [127,128,129]. Despite this, mouse models remain imperfect surrogates of the human pathology, and future work is required to consolidate and validate the role and function of miR-21 in liver pathologies and, in particular, its relevance in humans as a therapeutic target or biomarker for metabolic diseases and cancer. We do not believe that in vitro studies with cell lines will be helpful in this case because of the reasons discussed above, but combined analyses of animal models with the new generation of complex human liver organoids being currently developed should provide highly relevant information in the future. Additionally, different approaches, such as rescuing miR-21 expression with synthetic nucleotides in miR-21-deficient mice to investigate the outcomes for different diseases, should provide additional relevant data to confirm or disprove a tumor suppressor or an oncogenic role of miR-21 in specific diseases. Such an approach may also be useful to discern the specific role and function of the guide and passenger strand of miR-21, which might exert, as discussed above, different, or even opposite functions.

## 5. Conclusions

Based on the above considerations, understanding precisely the role of miR-21 in complex pathologies such as hepatic inflammation and cancer is challenging and requires further in-depth analyses. The present study, however, breaks the current dogma of miR-21 as a potent oncogene by showing that in the hepatic environment miR-21 can actually refrain liver cancer development and behaves as a tumor suppressor. These findings call for cautiousness when considering pharmacological miR-21 inhibitors as therapeutic weapons to treat liver cancer.

## Data Availability

The data presented in this study are available in this article (and in the Supplementary Materials).

## Supplementary Materials

The following are available online at www.mdpi.com/article/10.3390/cancers13194983/s1, Figure S1: RT-qPCR validation of miR-21 targets identified in RNAseq analysis, Figure S2: Gene Ontology enrichment analysis by biological processes of validated/predicted miR-21 targets upregulated under stress conditions, Figure S3: Kegg pathway gene enrichment analysis of validated/predicted miR-21 targets upregulated under stress conditions, Figure S4: CT-scan analyses of tumors number and size in LPTENKO and LPTENmiR21KO mice at 12 months of age, Figure S5: Immune cell infiltration and fibrosis in hepatic tissues of 12-month-old LPTENKO and LPTENmiR21KO mice, Figure S6: Loss of miR-21 does not affect apoptosis, Figure S7: MiR-21 deficiency leads to increased proliferation in hepatic tissue of LPTENKOmiR21KO mice, Figure S8: Most of miR-21 targets previously validated in literature are not modulated in the liver in vivo, Figure S9: DEN induces acute liver injury, inflammation, and immune cell recruitment, Figure S10: miR-21 deficiency alters NK cells activity in response to the carcinogen DEN, Figure S11: Loss of miR-21 in PTEN knockout hepatocytes is associated with increased expression of immune cells chemoattractants and recruitment of regulatory T cells (T reg), Figure S12: miR-21 is not always upregulated on tumoral tissues from patients diagnosed with HCC, Table S1: List of primers used and respective sequences, Table S2: List of antibodies used and respective references, Table S3: miR-21 loss promotes the hepatic deregulation of both ONCs, Ds, and TSs. Table S4: miR-21 loss also promotes hepatic deregulation of other genes that modulate metabolic and inflammatory processes.
