# Supplementary material for: Mir-21 Suppression Promotes Mouse Hepatocarcinogenesis
Source: Cancers (Basel). 2021 Oct 4;13(19):4983. doi: 10.3390/cancers13194983 (PMC8508272; doi:10.3390/cancers13194983)
Supplement: Supplementary file 1 [file cancers-13-04983-s001.zip › cancers-1383906-supplementary.pdf]

cancers

Supplementary Files

Mir-21 Suppression Promotes Mouse Hepatocarcinogenesis

Marta Correia de Sousa, Nicolas Calo, Cyril Sobolewski, Monika Gjorgjieva, Sophie Clément, Christine Maeder, Dobrochna Dolicka, Margot Fournier, Laurent Vinet, Xavier Montet, Jean-François Dufour, Bostjan Humar, Francesco Negro, Christine Sempoux and Michelangelo Foti.

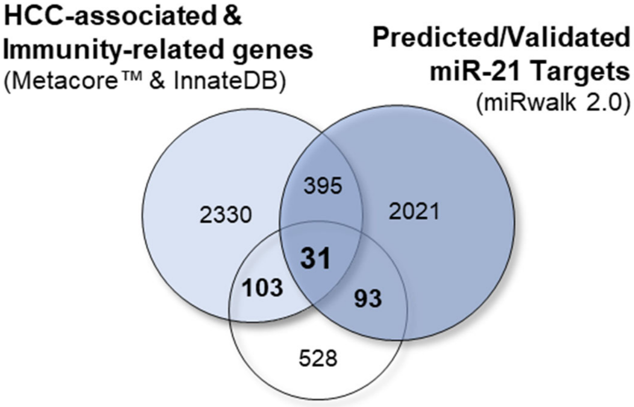

(**A**)

	Gene	log2FC	FC	P-value
Down-regulated genes	**Bdnf**	-1.65	0.32	0.0183
	**Ppp1r1b**	-1.45	0.37	0.0007
	**Hspb1**	-1.31	0.40	0.0001
	**Ptp4a1**	-1.14	0.45	0.0072
	**Lpin1**	-1.05	0.48	0.0156
	Per2	-1.03	0.49	0.0007
	Cd47	-0.69	0.62	0.0252
	Hpgd	-0.69	0.62	0.0165
	Tns1	-0.61	0.65	0.0283
Up-regulated genes	C8a	0.64	1.56	0.0387
	Pml	0.71	1.63	0.0228
	Selp	0.71	1.64	0.0424
	Pdcd4	0.73	1.66	0.0101
	**Mtor**	0.81	1.75	0.0044
	**Il6st**	0.86	1.81	0.0062
	**Appl1**	0.87	1.83	0.0126
	Cpd	0.87	1.83	0.0348
	Irak2	0.88	1.85	0.0049
	Marveld2	0.89	1.85	0.0086
	Col4a1	0.89	1.85	0.0072
	Nek6	0.96	1.94	0.0218
	**Cdc25a**	0.99	1.98	0.0124
	Cxcl9	1.03	2.05	0.0403
	**Lifr**	1.04	2.06	0.0002
	**Camkk2**	1.07	2.10	0.0074
	**Ngfr**	1.19	2.28	0.0379
	**Ccnd1**	1.30	2.47	0.0209
	**Sftpa1**	1.37	2.58	0.0140
	**Egfr**	1.37	2.59	0.0141
	**Tnfsf10**	1.46	2.75	0.0013
	**Cxcl10**	1.98	3.95	0.0034

(**B**)

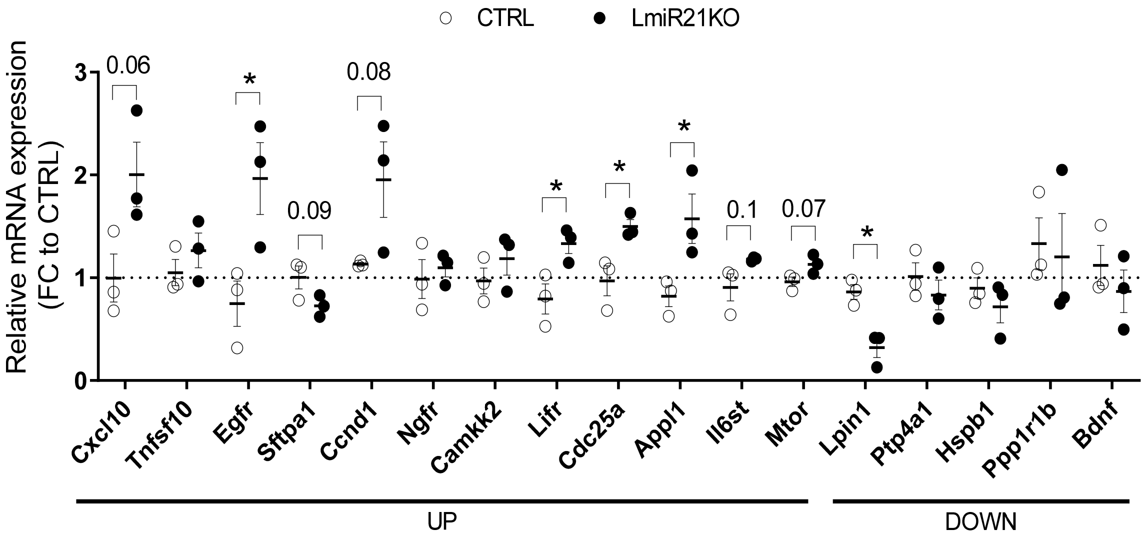

(**C**)

Figure S1. RT-qPCR validation of miR-21 targets potentially contributing to hepatocarcinogenesis. (A) List of deregulated genes (DEG) (RNAseq analysis) in hepatic tissues of LmiR21KO livers as compared to CTRL mice (see also Figure 1). DEG were cross-referenced with a list of genes associated with hepatocellular carcinoma (HCC), immunity and predicted/validated targets of miR-21-5p/-3p extracted from commercial/public databases (Metacore™ and InnateDB) and miRwalk 2.0, respectively. 31 potential candidates were identified with this approach. (B) Relative expression of the 31 candidates in the RNA-seq analysis of explanted liver tissues LmiR21KO mice fed an obesogenic diet for 3 weeks (n=3 per group). Fold-change (FC) to Control group and corresponding p-value is indicated. Upregulated genes are represented in green and downregulated genes are represented in pink. (C) RT-qPCR validation analysis of 16/31 potential candidates (genes in bold in B) in the same hepatic tissues where RNA-seq analysis was performed. Unpaired t-test with Welch's correction was used for comparison between two groups. * p-value <0.05, ** p-value < 0.01, *** p-value < 0.001, **** p-value <0.0001.

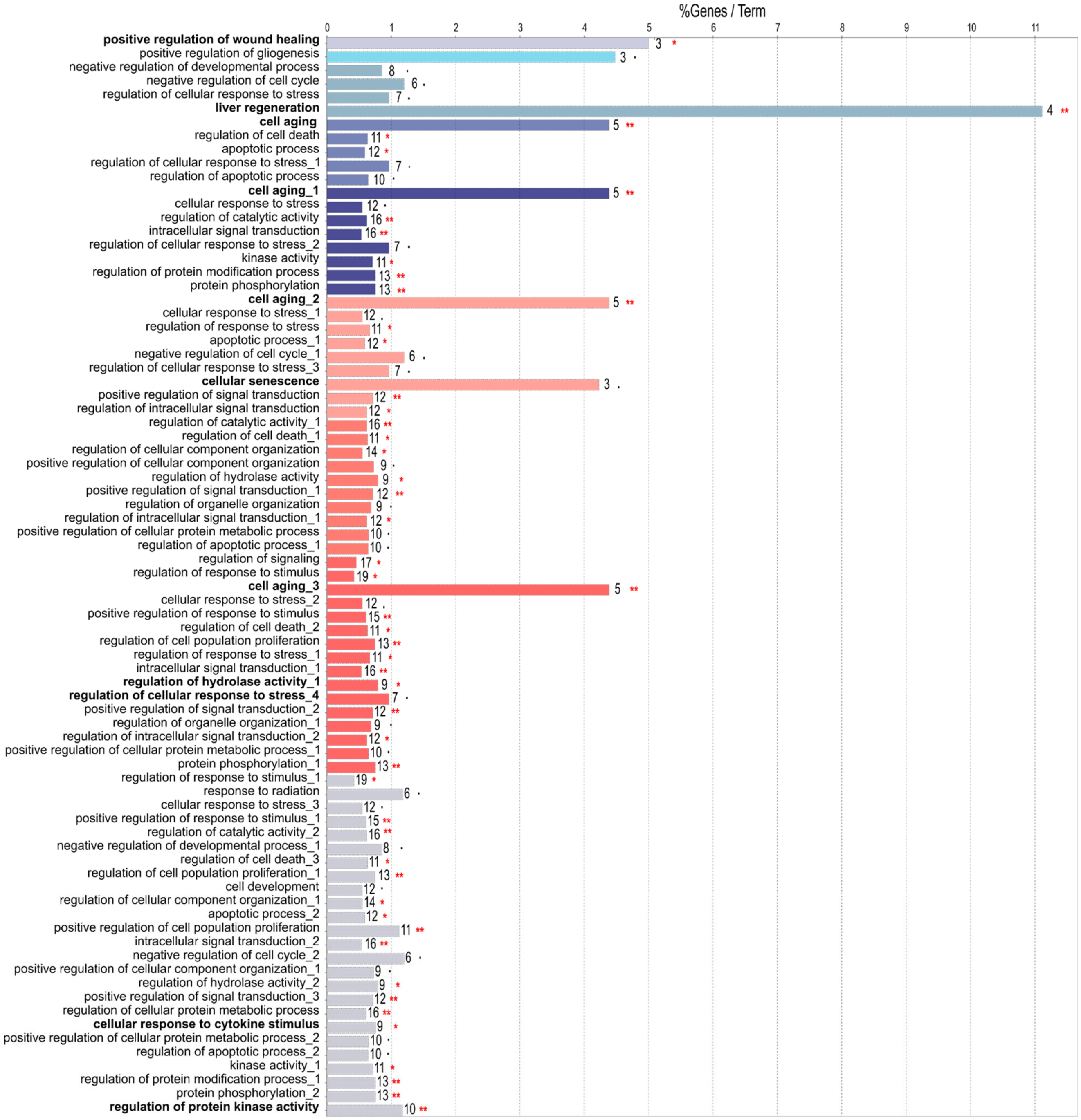

Figure S2. MiR-21 targets upregulated under stress conditions are involved in cancer-related biological processes. ClueGO+CluePedia (Cytoscape) analysis of deregulated genes (upregulated DEGs) potentially targeted by miR-21 (illustrated in Figure 1C) highlights functionally grouped network of biological processes (clusters of terms) with putative interactions between them, based on their kappa score level (0.45). Each cluster of terms is represented by a specific color. The number of genes (from input) involved in a specific term, as well as the term's enrichment significance within the cluster (adjusted p-value ≤0.10), are indicated in the graph. Two-sided hypergeometric test with Bonferroni step down correction was used to calculate enrichment significance. * p-value <0.05, ** p-value < 0.01, *** p-value < 0.001, **** p-value <0.0001.

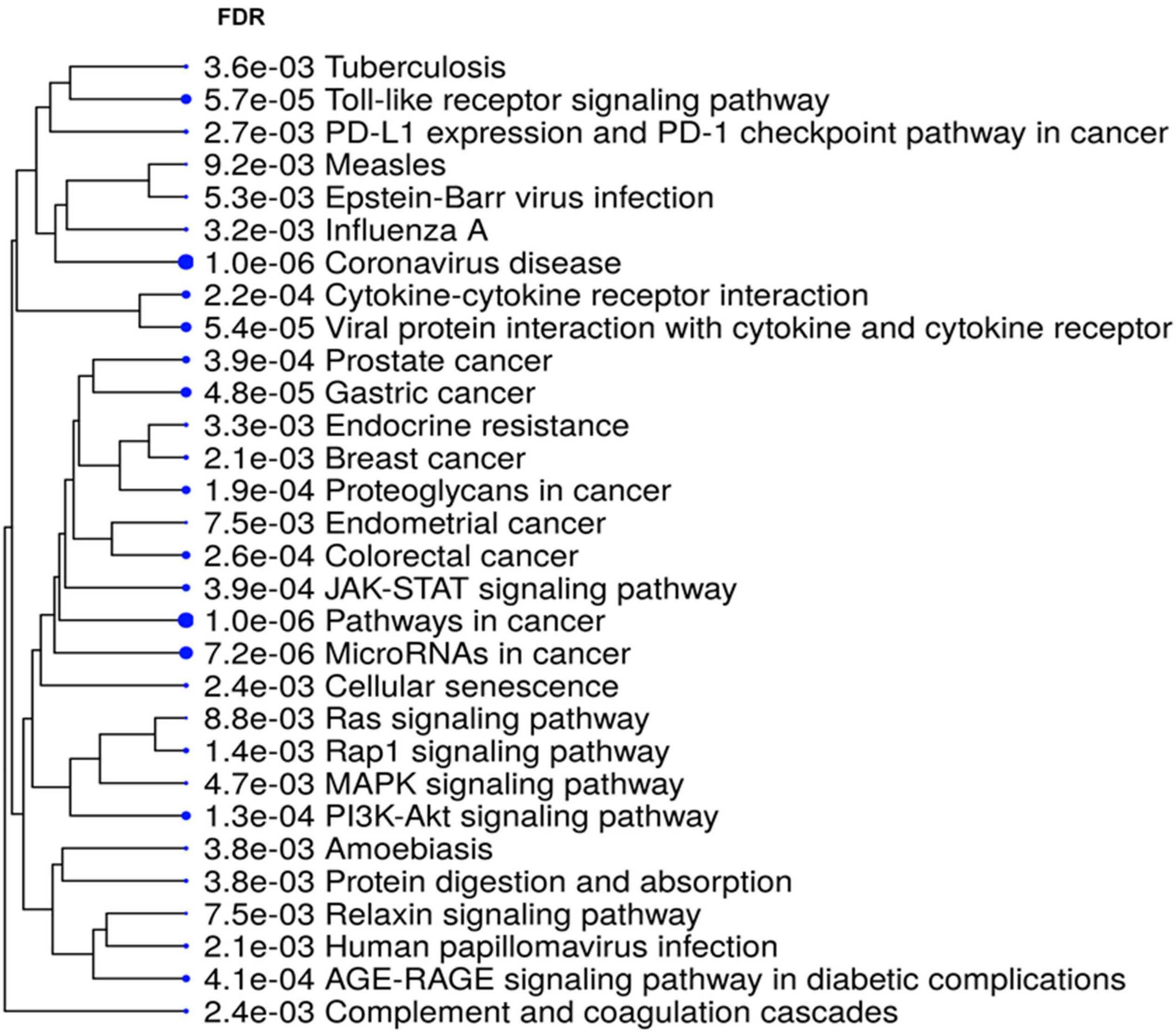

Figure S3. Gene Ontology enrichment analysis of potential miR-21 targets upregulated under stress conditions. Gene Ontology (GO) enrichment analysis based on Kyoto Encyclopedia of Genes and Genomes (KEGG) pathways was performed on upregulated genes identified in the transcriptome of LmiR21KO challenged with high-fat diet (illustrated in Figure 1C-E). Pathways with False Discovery Rate (FDR) < 5% are represented.

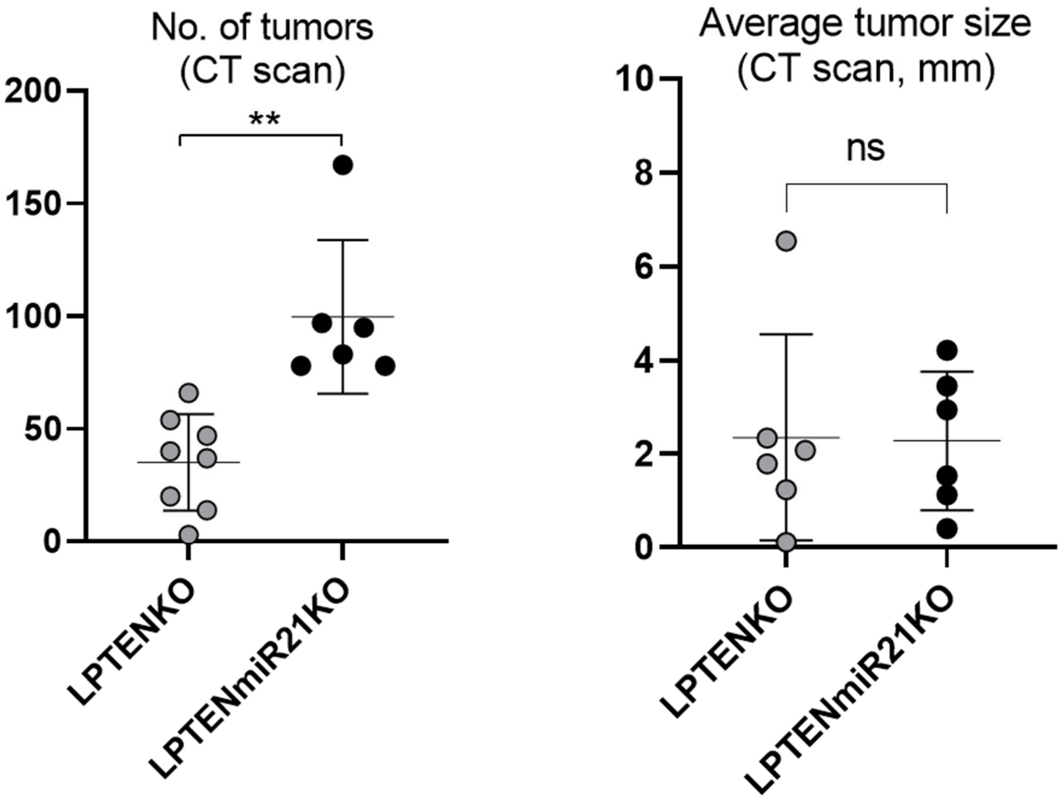

Figure S4. CT-scan analyses of tumoral nodules in hepatocyte-specific PTEN and PTEN/miR-21 knockout mice. (A) Number and **(B)** average tumor size of individual tumors per mouse identified and calculated by CT scan imaging of one-year old LPTENKO (n=6-8) and LPTENmiR21KO (n=6) mice. Data is analyzed via the OsiriX software and represented as mean ± SD. Outliers test was performed using ROUT method (Q=1%). Unpaired t-test with Welch's correction was used for comparison between two groups. * p-value <0.05, ** p-value < 0.01, *** p-value < 0.001, **** p-value <0.0001.

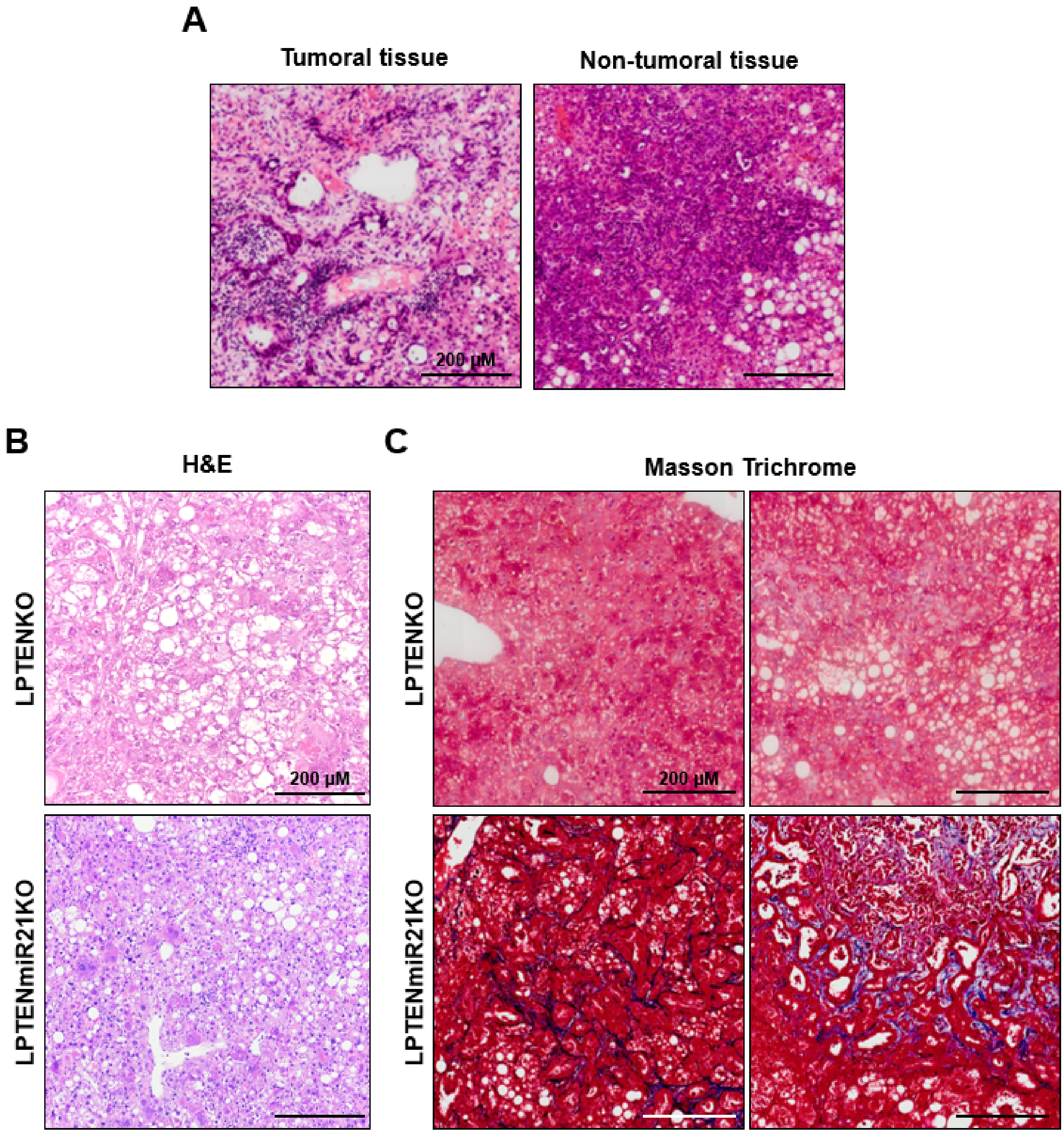

Figure S5. Hepatocyte-specific deletion of miR-21 in LPTENKO mice promotes inflammation and fibrosis. (A) Severe immune infiltration (dark and dense nuclei typical from immune cells) was observed in the livers of LPTENmiR21KO mice, not only in non-tumoral tissue (left) but also surrounding tumoral masses (right) (12 months old mice). **(B)** Analysis of hematoxylin-eosin (H&E) stained sections of livers from LPTENKO and from LPTENmiR21KO mice revealed the occurrence of steatohepatitis in both models, with an exacerbation of the phenotype on the latter. (C) Fibrosis was observed in LPTENmiR21KO mice but not in LPTENKO, and mainly in the center of tumoral masses of mixed origin - Masson-trichrome staining, magnification X40. Evaluation of steatosis , inflammation and fibrosis was performed by an expert liver pathologist (Prof. Christine Sempoux, Institute of Pathology, Lausanne University Hospital, University of Lausanne, Lausanne, Switzerland).

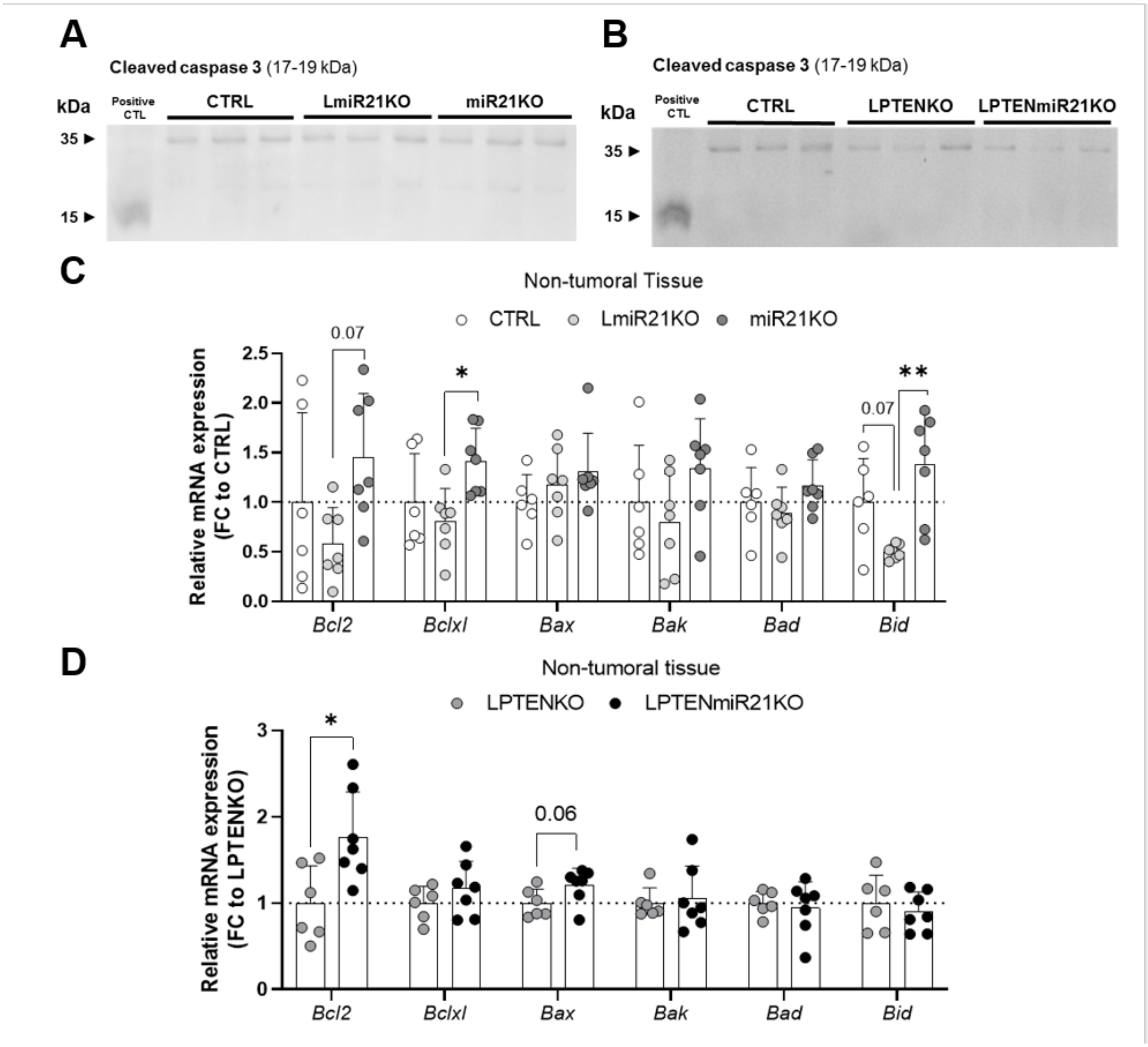

Figure S6. Apoptosis in mouse hepatic tissues is not affected by miR-21 deficiency. Western blot analysis of cleaved caspase 3 in the liver tissues of **(A)** DEN-treated 11 months-old control (CTRL, n=6), hepatocyte-specific (LmiR21KO, n=6) and total miR-21 (miR21KO, n=8) knockout mice, and **(B)** 12 months-old wild-type (CTRL, n=5), hepatocyte-specific PTEN knockout (LPTENKO, n=6) and hepatocyte-specific PTEN-miR21 knockout (LPTENmiR21KO, n=8) mice. HepG2 cells treated with staurosporin (1 μmol for 48 hours) were used as positive control for apoptosis (first lane). RT-qPCR analysis of apoptosis-related markers *(Bcl2, Bclxl, Bax, Bak, Bad, Bid)* in the livers of **(C)** DEN-treated 11 months-old control (CTRL, n=6), hepatocyte-specific (LmiR21KO, n=7) and total miR-21 (miR21KO, n=7) knockout mice, and **(D)** 12 months-old hepatocyte-specific PTEN knockout (LPTENKO, n=6) and hepatocyte-specific PTEN-miR21 knockout (LPTENmiR21KO, n=7) mice. Data from DEN-treated LmiR21KO and miR-21KO mice are represented as mean± SD fold-change (FC) to DEN treated CTRL mice. Data of LPTENmiR21KO mice is represented as mean± SD fold-change (FC) to LPTENKO. Data that failed normality tests was submitted to log10-transformation prior to statistical analysis. Outliers test was performed using ROUT method (Q=1%). Unpaired t-test with Welch's correction was used for comparison between two groups. One-way ANOVA with Holm-Sidak's correction was used for comparison between more than two groups. * p-value <0.05, ** p-value < 0.01, *** p-value < 0.001, **** p-value <0.0001.

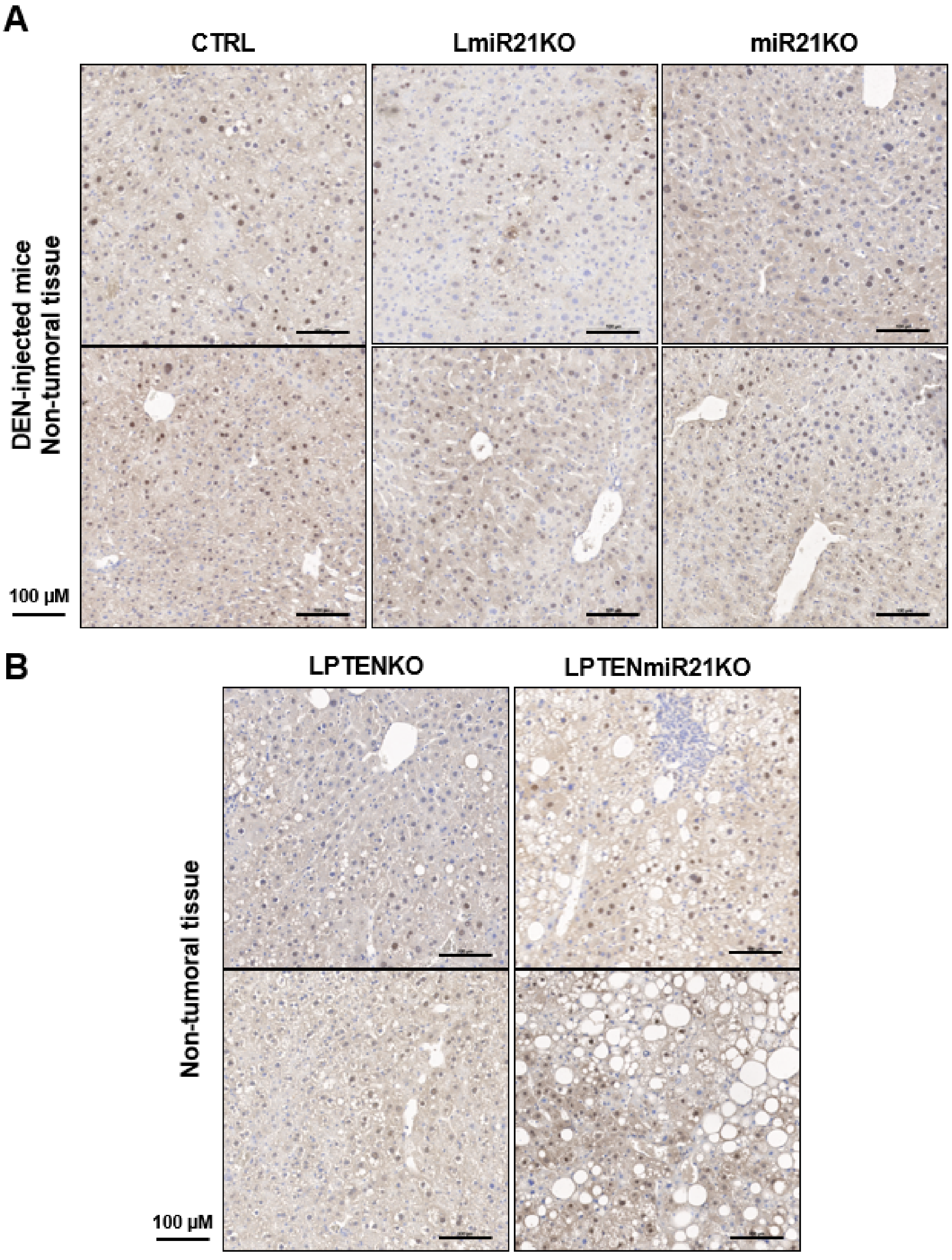

Figure S7. Ki67 immunohistological staining of liver sections from controls and miR-21 deficient mice. Representative immunohistochemical staining of the Ki67 proliferation marker in liver sections of 11 months-old DEN-treated mice (CTRL, LmiR21KO and miR21KO mice) and **(B)** 12 months-old LPTENKO and LPTENmiR21KO mice.

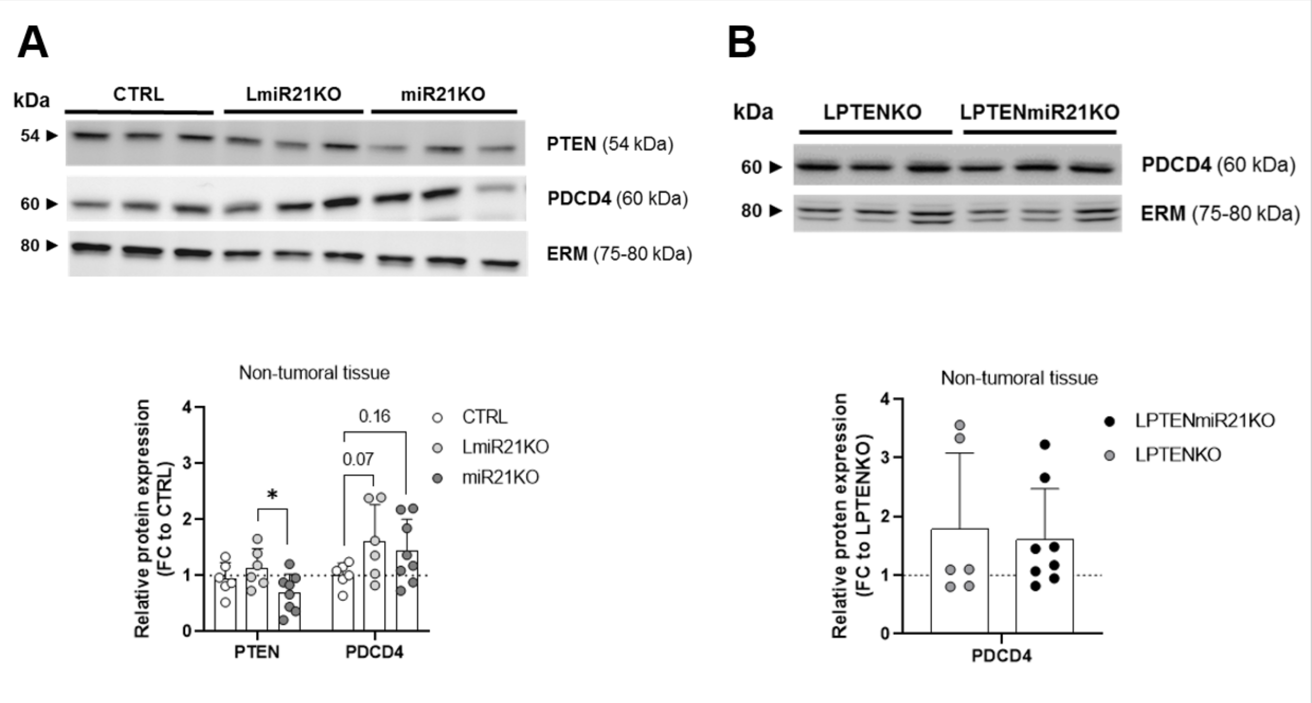

Figure S8. miR-21 deficiency does not affect the expression of PTEN and PDCD4 in hepatic tissues. (A) Representative Western blot (top panel, 3 mice per group) and quantifications (bottom panel) of PTEN and PDCD4 in the hepatic tissues of 11 months-old control , hepatocyte-specific and total miR-21 knockout mice injected with DEN. Quantifications were performed on Western blot analysis of n=6 CTRL mice, n=6 LmiR21KO and n=8 miR21KO. Data from DEN-injected miR-21 KO mice is represented as mean± SD fold-change to DEN-treated CTRL mice. **(B)** Representative Western blot (top panel, 3 mice per group) and quantifications (bottom panel) of PDCD4 in the hepatic tissues of 12 months-old hepatocyte-specific PTEN and PTEN-miR21 knockout mice. Quantifications were performed on Western blot analysis of n=5 LPTENKO and n=8 LPTENmiR21KO. Data of LPTENKO and LPTENmiR21KO mice is represented as mean± SD FC to LPTENKO. Data that failed normality tests was submitted to log10-transformation prior to statistical analysis. Outliers test was performed using ROUT method (Q=1%). Unpaired t-test with Welch's correction was used for comparison between two groups. One-way ANOVA with Holm-Sidak's correction was used for comparison between more than two groups. * p-value <0.05, ** p-value < 0.01, *** p-value < 0.001, **** p-value <0.0001.

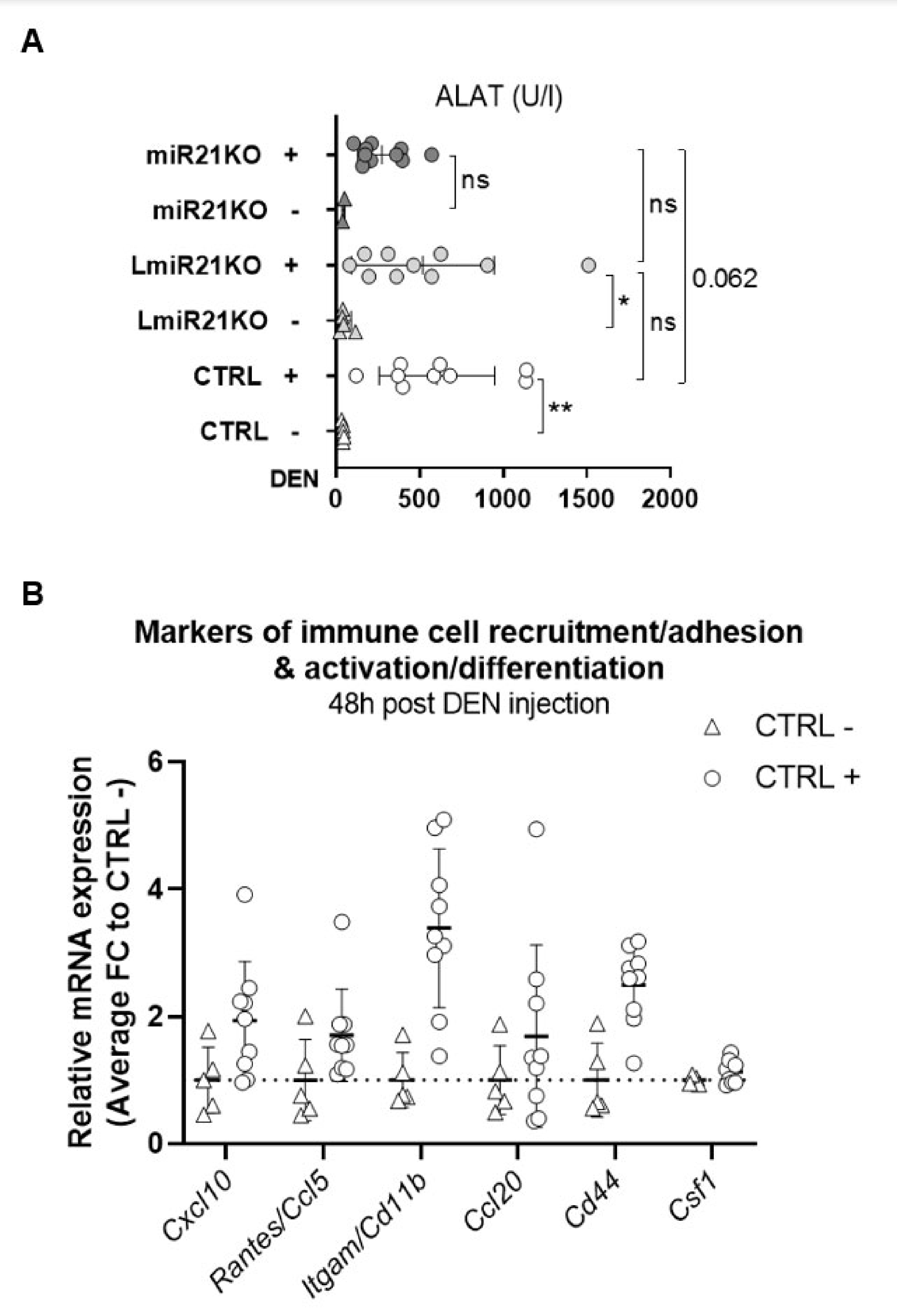
A
ALAT (U/l)
miR21KO +
miR21KO -
LmiR21KO +
LmiR21KO -
CTRL +
CTRL -
DEN
0
500
1000
1500
2000
ns
ns
*
ns
0.062
**
B
Markers of immune cell recruitment/adhesion & activation/differentiation
48h post DEN injection
CTRL -
CTRL +
Relative mRNA expression (Average FC to CTRL -)
0
2
4
6
Cxcl10
Rantes/Ccl5
Itgam/Cd11b
Ccl20
Cd44
Csf1

Figure S9. DEN exposure leads to acute liver injury, inflammation and immune cell recruitment in mice. (A) Plasma alanine-aminotransferase (ALAT) levels in 2 months-old mice 48h post-diethylnitrosamine injection (DEN, 100 mg/kg of body weight i.p.) or with NaCl from CTRL (**DEN +** n=9, **DEN -** n=5), LmiR21KO (**DEN +** n=10, **DEN -** n=5) and miR21KO (**DEN +** n=10, **DEN -** n=2) groups. Data is represented as mean ± SD and outliers test was performed using ROUT method (Q=1%). **(B)** Relative mRNA expression of inflammatory markers involved in immune cell recruitment, adhesion, activation and differentiation in hepatic tissues of 2 months-old mice 48h post-DEN injection (100 mg/kg of body weight, CTRL: **DEN +** n=9, **DEN -** n=5). Data from DEN-injected miR-21 KO mice is represented as mean± SD fold-change to CTRL mice (**DEN -**, NaCl injected). One-way ANOVA with Holm-Sidak's correction was used for comparison between more than two groups. * p-value <0.05, ** p-value < 0.01, *** p-value < 0.001, **** p-value <0.0001.

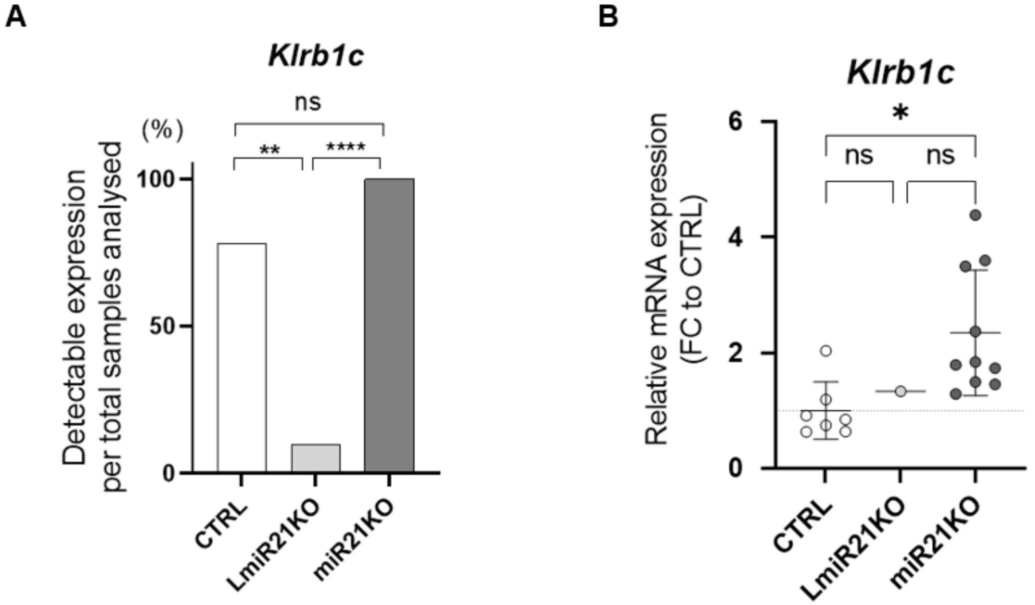

Figure S10. Hepatocyte-specific or total miR-21 deletion impacts NK cells activity in response to DEN exposure (A) Detectable expression of the *Klrb1c* marker of Natural Killer (NK) cells cytotoxicity activity in hepatic tissues of 2 months-old mice 48h post-DEN injection . Data is represented as percentage of samples with detectable expression *versus* total number of analysed samples (CTRL n=9, LmiR21KO n=10, miR21KO n=10). **(B)** Relative mRNA expression of *Klrb1c* gene in hepatic tissues of 2 months-old mice 48h post-DEN injection in samples with detectable levels of *Klrb1c* expression. Data from DEN-injected miR-21 KO mice is represented as mean± SD fold-change to CTRL mice (miR21fx treated with DEN). Chi-Square Test was used to evaluate the independence of categorical variables (percentage of samples with detected expression). One-way ANOVA with Holm-Sidak's correction was used for comparison between more than two groups. * p-value <0.05, ** p-value < 0.01, *** p-value < 0.001, **** p-value <0.0001.

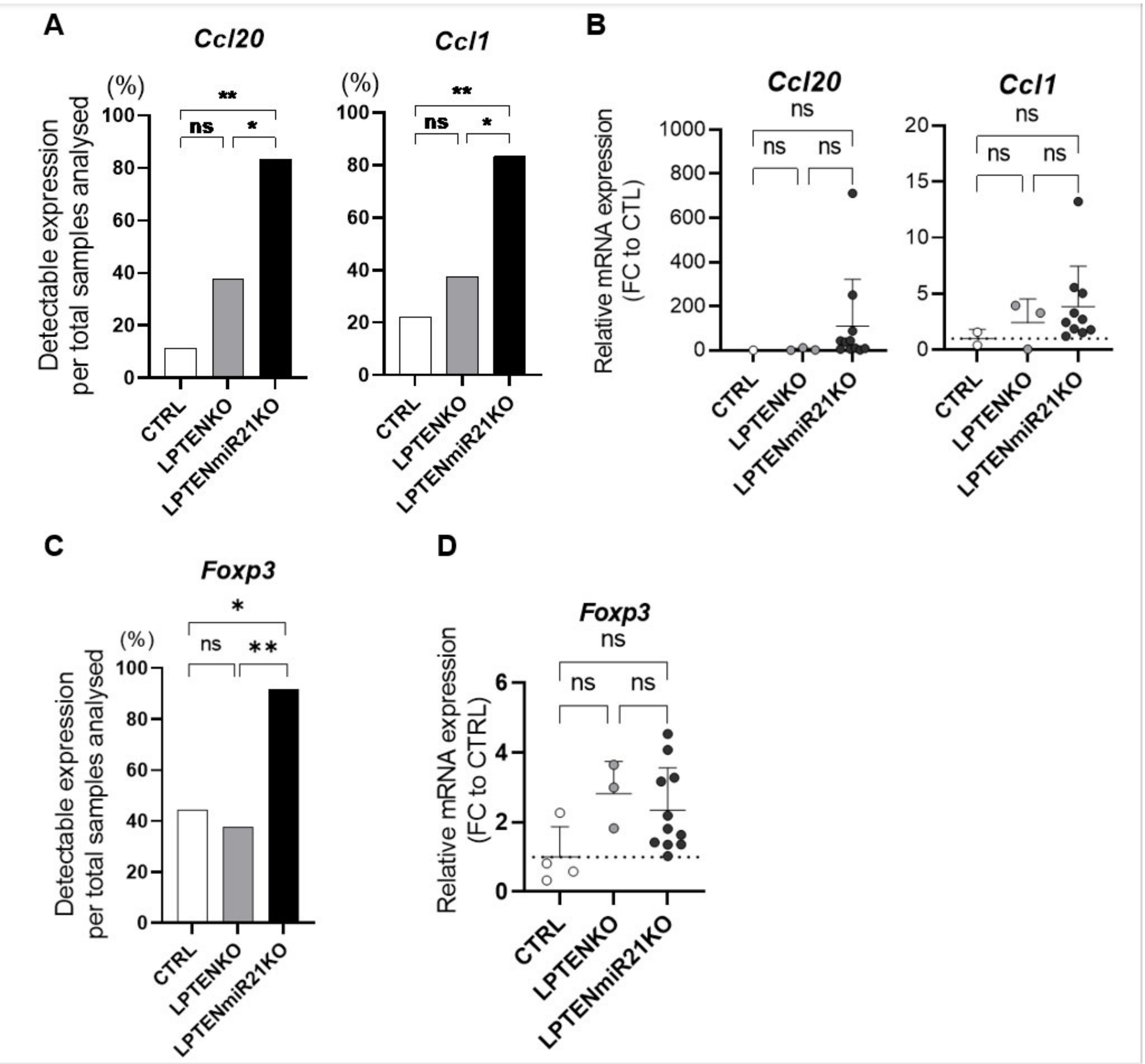

Figure S11. Loss of miR-21 in PTEN knockout hepatocytes fosters a pro-inflammatory environment and the infiltration of regulatory T cells (T reg). (A) Percentage of samples from 7 months-old mice with detectable expression by RT-qPCR of *Ccl20* and *Ccl1* markers of immune cell recruitment. (B) *Ccl20* and *Ccl1* relative mRNA expression in 7 months-old mice . (C) Percentage of samples from 7 months-old mice with detectable expression by RT-qPCR of *Foxp3* a marker of regulatory T cells. (D). *Foxp3* relative mRNA expression in 7 months-old mice . Analysis were performed on hepatic tissues of 7 months-old mice (CTRL: n=9, LPTENKO: n=8; LPTENmiR21KO mice: n=12). Detectable expression data is represented as percentage of samples with detected expression versus total number of analysed samples. Relative mRNA expression is represented as mean ± SD of the fold-change to CTRL mice. Chi-Square Test was used to evaluate the independence of categorical variables (percentage of samples with detected expression). P-value is indicated in the graphs. One-way ANOVA with Holm-Sidak's correction was used for comparison between more than two groups.

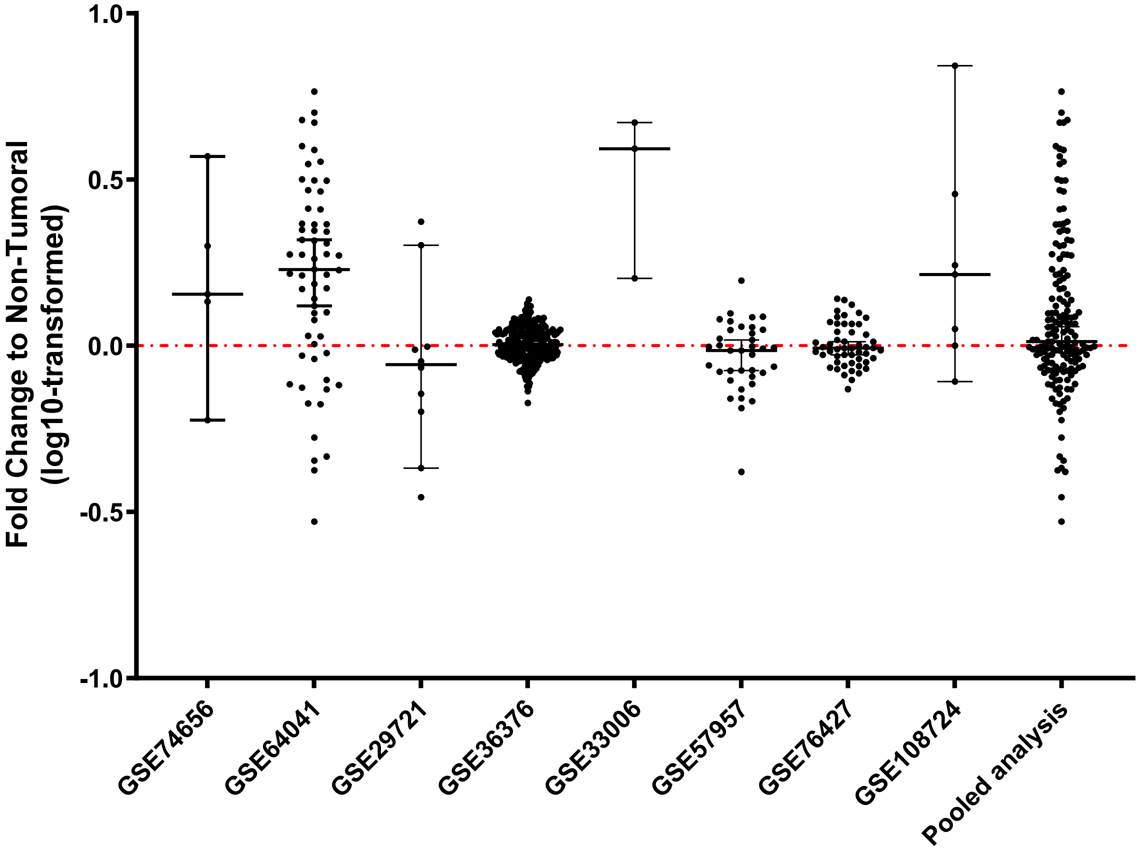

Figure S12. MiR-21 expression in human HCC samples *versus* non-tumoral liver tissue. The graph represents fold change of miR-21 expression in human hepatocellular carcinoma biopsies as reported to the associated non-tumoral hepatic tissue of the donor. Data were obtaimed from 8 different GEO datasets (GSE74656, GSE64041, GSE29721, GSE36376, GSE33006, GSE57957, GSE76427, GSE108724). The last column represents a pooled analysis of all of the samples analysed from the 8 GEO datasets. Fold change is represented as mean +/-SD for each GEO dataset.

Table S1. List of Primers used and respective sequences

Gene	Forward (5' --> 3')	Reverse (5' --> 3')	Species
Appl1	AGCCAGTGACCCTTTATATCTGC	AGGTATCCAGCCTTTCGGGTT	Mouse
Arg1	ATGGGCAACCTGTGTCCTTT	TCTACGTCTCGCAAGCCAAT	Mouse
Bad	GGAAGACGCTAGTGCTACAGA	GAGCCTCCTTTGCCCAAGTTT	Mouse
Bak	CCCAGGACACAGAGGAGGTC	GCCCAACAGAACCACACCAAAA	Mouse
Bax	CGGCGAATTGGAGATGAACTG	GCAAAGTAGAAGAGGGCAACC	Mouse
Bcl2	TACCGTCGTGACTTCGCAGAG	GGCAGGCTGAGCAGGGTCTT	Mouse
Bclxl	AGGCAGGCGATGAGTTTGAAC	GAACCACACCAGCCACAGTCA	Mouse
Bid	GCCAAGCACATCACAGACCTG	GAAGACATCACGGAGCAAAGAT	Mouse
Camkk2	TCATGTGTCTCTAGCCAGCC	TGACCACGATGAAGGATTCCAT	Mouse
Ccl1	GGCTGCCGTGTGGATACAG	AGGTGATTTTGAACCCACGTTT	Mouse
Ccl20	ACTGTTGCCTCTCGTACATACA	GAGGAGGTTCACAGCCCTTTT	Mouse
Ccl22	CTTGCTGTGGCAATTCAGACC	CTTGCTGTGGCAATTCAGACC	Mouse
Ccnb1	AAGGTGCCTGTGTGTGAACC	GTCAGCCCCATCATCTGCG	Mouse
Ccnd1	TGACTGCCGAGAAGTTGTGC	CTCATCCGCCTCTGGCATT	Mouse
Cd11c	CAGCTATACTTTACAAGGCTGGTTTCT	CCGTCTGAGACAAACTGTCCATT	Mouse
Cd14	CTCTGTCCTTAAAGCGGCTTAC	GTTGCGGAGGTTCAAGATGTT	Mouse
Cd163	ATGGGTGGACACAGAATGGTT	CAGGAGCGTTAGTGACAGCAG	Mouse
Cd19	AGTGATTGTCAATGTCTCAGACC	CTCCCCACTATCCTCCACGTT	Mouse
Cd335	ATGCTGCCAACACTCACTG	GATGTTCACCGAGTTTCCATTTG	Mouse
Cd38	TCTCTAGGAAAGCCCAGATCG	GTCCACACCAGGAGTGAGC	Mouse
Cd3e	ATGCGGTGGAACACTTTCTGG	GCACGTCAACTCTACACTGGT	Mouse
Cd4	TCCTAGCTGTCACTCAAGGGA	TCAGAGAACTTCCAGGTGAAGA	Mouse
Cd44	ACTTTGCCTCTTGCAGTTGAG	TTTCTCCACATGGAATACACCTG	Mouse
Cd80	TGCTGCTGATTCGTCTTTCAC	GAGGAGAGTTGTAACGGCAAG	Mouse
Cd8a	AAGAAAATGGACGCCGAACTT	AAGCCATATAGACAACGAAGGTG	Mouse
Cdc25a	ACAGCAGTCTACAGAGAATGGG	GATGAGGTGAAAGGTGTCTTGG	Mouse
CDC25A	GTGAAGGCGCTATTTGGCG	TGGTTGCTCATAATCACTGCC	Human
Cdk1	GTCCGTCGTAACCTGTTGAG	TGACTATATTTGGATGTCGAAG	Mouse
Col4a1	TCCGGGAGAGATTGGTTTCC	CTGGCCTATAAGCCCTGGT	Mouse
Cpd	CTGAGGCGACCACGACAAC	CTGCCGATGCTGAAGAGAC	Mouse
Csf1	ATGGACACCTGAAGGTCCTG	GTTAGCATTGGGGGTGTTGT	Mouse
Cxcl10	CCAAGTGCTGCCGTCATTTTC	GGCTCGCAGGGATGATTTCAA	Mouse
Cxcl9	TCCTTTTGGGCATCATCTTCC	TTTGTAGTGGATCGTGCCTCG	Mouse
Egfr	GCCATCTGGGCCAAAGATACC	GTCTTCGCATGAATAGGCCAAT	Mouse
Egr2	GCCAAGGCCGTAGACAAAATC	CCACTCCGTTCATCTGGTCA	Mouse
Fasl	TCCGTGAGTTCACCAACCAAA	GGGGGTTCCCTGTTAAATGGG	Mouse
Foxp3	CCCATCCCCAGGAGTCTTG	ACCATGACTAGGGGCACTGTA	Mouse
Fpr2	TTACACCACAGGAACCGAAGA	CAGCACACCAAGGAAGAAAGTG	Mouse
Gak	CTGCCCACCAGGCATTTG	CCATGTCACATACATATTCAATGTACCT	Mouse
Gpr18	TCAGAGCTCAGCAGACCAAC	TGGTATGTAGAACCTCCTGTCTG	Mouse
Havcr2 Tim3	TCAGGTCTTACCCTCAACTGTG	GGGCAGATAGGCATTTTTACCA	Mouse
Hbp1	TGGCTTGCTCACTGTAGAGTGC	CAGGAGGTAGACATACGTCACC	Mouse
Hpgd	CAACAATGCAGGCGTGAACA	GCGTGTGAATCCGATGATGC	Mouse
Ifng	ACAATGAACGCTACACACTGCAT	TGGCAGTAACAGCCAGAAACA	Mouse
Il10	CTTTCAAACAAAGGACCAGC	CCAAGTAACCCTTAAAGTCCT	Mouse
Il17a	TTTAACTCCCTTGGCGCAAAA	CTTTCCCTCCGCATTGACAC	Mouse
Il1b	GACAACTGCACTACAGGC	CATGGAGAATATCACTTGTTGG	Mouse
Il6	AGTTGCCTTCTTGGGACTGAT	TCCACGATTTCCCAGAGAAC	Mouse
Il6st	CTTGCATTGTGAATGAGGGGA	TGGGCATGTAGCTGACCATAC	Mouse
Itgam	ATGGACGCTGATGGCAATACC	TCCCCATTCACGTCTCCCA	Mouse
Ki67	TGGTCACCATCAAGCGGAG	AGGCAGCTGGATACGAATGT	Mouse
Klrb1c	GACACAGCAAGTATCTACCTCGG	TCAGAGCCAACCTGTGTGAACG	Mouse
Marveld2	ACCCGTCGTTGTCATCGAAAA	TCACGATAGGCTTCGGAATGT	Mouse
Mtor	CAGTTCGCCAGTGGACTGAAG	GCTGGTCATAGAAGCGAGTAGAC	Mouse
Nek6	ACATGCCTCATGGAGGGAG	GCCAGTGAGCAGCGAAAAG	Mouse
P21	ACAAGAGGCCCAGTACTTCC	AGAAATCTGTCAGGCTGGTCT	Mouse
P27	CGAGTTCTACTACAGGCCCC	GACGAGTCAGGCATTTGGTC	Mouse
Pcna	CCTGTGCAAAGAATGGGGTG	TCTCTATGGTTACCGCCTCC	Mouse
Pdcd4	CGGTTAGAAGTGGAGTTGC	ACAGTTCTCCTGGTCGTC	Mouse
PPIA	ATGGTCAACCCCACCGTGT	TCTGCTGTCTTTGGGACCTTGTC	Human
Ppia	CAAATGCTGGACCAAACACAA	GCCATCCAGCCATTCAGTCT	Mouse

Gene	Forward (5' --> 3')	Reverse (5' --> 3')	Species
Per2	GAAAGCTGTCACCACCATAGAA	AACTCGCACTTCCTTTTCAGG	Mouse
Ppara	ACGATGCTGTCCTCCTTGATG	GTGTGATAAAGCCATTGCCGT	Mouse
Pten	ACACCGCCAAATTTAACTGC	TACACCAGTCCGTCCCTTTC	Mouse
Rantes/Ccl5	CTCACCATATGGCTCGGA	TTCCTTCGAGTGACAAACAC	Mouse
Rps9	GACCAGGAGCTAAAGTTGATTGGA	TCTTGGCCAGGGTAAACTTGA	Mouse
Selp	CATCTGGTTCAGTGCTTTGATCT	ACCCGTGAGTTATTCCATGAGT	Mouse
Sftpa1	GAGGAGCTTCAGACTGCACTC	AGACTTTATCCCCCACTGACAG	Mouse
Spry2	TGAAAGACTCCACGGTCTGC	AGCTGACAGTGCTGATGGAC	Mouse
Srp72	CACCCAGCAGACAGACAAACTG	GCACTCATCGTAGCGTTCCA	Mouse
Tgfb	CAACATGTGGAACTCTACCAG	TGTATTCCGTCTCCTTGGT	Mouse
Timp3	ACACGGAAGCCTCTGAAAGT	TCATACACGCGCCCTGTCA	Mouse
Tnfa	GGCTGCCCCGACTACGT	GACTTTCTCCTGGTATGAGATAGCAA	Mouse
miR-21	CGCTAGCTTATCAGACTGATG	GAGGTATTCGCACCAGAGGA (universal miRNA reverse primer)	Human and Mouse
miR-122	GGCTGTGGAGTGTGACAATG	GAGGTATTCGCACCAGAGGA (universal miRNA reverse primer)	Human and Mouse
miR-16	ACAGCCTAGCAGCACGTAAAT	GAGGTATTCGCACCAGAGGA (universal miRNA reverse primer)	Human and Mouse

Table S2. – List of antibodies used for Western blot analysis and immunohistochemistry.

**Protein**	Antibody production (Catalog no.)	Dilution
Ki67	Millipore (AB9260)	1:150 (IHC)
Phospho-ERK1/2	Cell signalling (#9101)	1:1000 (WB)
ERK 1/2	Cell signalling (#9102)	1:1000 (WB)
Phospho-P38 (Thr180/Tyr182)	Cell signalling (#4511)	1:1000 (WB)
P38	Cell signalling (#8690)	1:1000 (WB)
Phospho-JNK	Cell signalling (#9251)	1:1000 (WB)
JNK	Cell signalling (#9252)	1:1000 (WB)
Phospho-STAT3	Cell signalling (#9145)	1:1000 (WB)
STAT3	Cell signalling (#12640)	1:1000 (WB)
Phospho-AKT (Ser473)	Cell signalling (#9271)	1:1000 (WB)
AKT	Cell signalling (#9272)	1:1000 (WB)
Phospho-YAP (Ser127)	Cell signalling (#13008)	1:1000 (WB)
YAP	Cell signalling (#14074)	1:1000 (WB)
ERM	Cell signalling (#3142)	1:1000 (WB)
PTEN	Cell signalling (#9559)	1:1000 (WB)
PDCD4	Cell signalling (#9535)	1:1000 (WB)
Cleaved Caspase 3	Cell signalling (#9661)	1:1000 (WB)
Goat Anti-rabbit IgG-HRP	BioRad (#1706515)	1:10000 (WB)

Supplementary Table 3. List of potential miR-21 target genes deregulated in the transcriptomic analysis performed on hepatic tissues from hepatocyte-specific miR-21 knockout (LmiR21KO) challenged with high-fat diet for 3 weeks. Genes deregulated in the transcriptomic analysis performed on hepatic tissues of LmiR21KO (Figure 1) were classified as oncogenes (ONC, in black), tumor supressors (TS, in blue) or drivers (D, in green) based on literature screening (source: CancerMine database). Non-classified genes (NC, in grey) and genes with double functions (marked with *) are also indicated.. Genes up- or downregulated in LimiR21KO mice are indicated with red upward or grey downward arrows, respectively.

Table S3. – Classification of 124 genes of interest as ONC, D or TS (1/4).

**Genes deregulated in LImir21KO targeted by miR-21** *(predicted and validated)*	**Genes classified as** *Sources: CancerMine*	**Expression in LImiR-21KO transcriptomic** (p-value<0.05)
AACS	NC	↓
ABCA2	NC	↑
ABCB1	NC	↑
ABCC3	TS	↓
ACP3	NC	↑
ADAMTS9	TS	↓
ADH1	NC	↓
ALB	D	↓
ANGPTL4	TS	↓
APPL1	NC	↑
AR	D	↑
ARAP2	NC	↑
ARHGEF10L	NC	↑
ATF5	ONC	↑
ATP10D	NC	↓
ATP7B	ONC	↑
BCL2L1	ONC	↓
BDNF	ONC	↓
BIRC5	ONC	↓
BMP7	ONC	↓
BRI3	NC	↓
C6	NC	↑
C8A	NC	↑
CAMKK2	D	↑
CCND1	ONC	↑
CD36	D	↓
CD47*	D/ONC	↓
CD5L	NC	↑
CDC25A	ONC	↑
CDH1*	ONC/TS	↓
CES2A	NC	↓
CFHR1/2	NC	↓
CHORDC1	D	↓
CISH	ONC	↓
COL12A1	ONC	↑
COL15A1	NC	↑
COL4A1	NC	↑

Table S3. – Classification of 124 genes of interest as ONC, D or TS (2/4).

**Genes deregulated in Llmir21KO targeted by miR-21** *(predicted and validated)*	**Genes classified as** *Sources: CancerMine*	**Expression in LlmiR-21KO transcriptomic** (p-value<0.05)
COL4A2	NC	↑
COMT	ONC	↑
CPD	D	↑
CXCL10	D	↑
CXCL14	TS	↑
CXCL9	NC	↑
CYP17A1	NC	↑
CYP2D34	NC	↓
CYP2E1	NC	↓
CYP7A1	NC	↓
DACT2	TS	↑
DAGLA	TS	↑
DDX60	NC	↑
DNMT3B*	ONC/TS	↑
DUSP1*	ONC/TS	↓
EFNA1	NC	↑
EGFR	ONC	↑
ESR1*	ONC/TS	↑
ESRRG	TS	↓
FGB	NC	↑
FGFR2	ONC	↑
FOXO1*	ONC/TS	↓
FRRS1	NC	↑
FST	ONC	↑
FUCA2	NC	↑
FURIN*	D/ONC/TS	↑
FZD6	D	↑
GJB2	TS	↑
GRAMD4	NC	↑
GTF3C1	NC	↑
HIPK2	TS	↑
HNF1B	TS	↑
HPGD	TS	↓
HSPA1B*	D/ONC	↓
HSPH1	NC	↓
IL22RA1	NC	↑
IL6ST*	TS/ONC	↑

Table S3. – Classification of 124 genes of interest as ONC, D or TS (3/4).

**Genes deregulated in Llmir21KO targeted by miR-21** *(predicted and validated)*	**Genes classified as** *Sources: CancerMine*	**Expression in LlmiR-21KO transcriptomic** (p-value<0.05)
IRAK2*	ONC/TS	↑
IRAK4	ONC	↑
IRF5	TS	↑
KRT18*	ONC/TS	↑
LGALS1	ONC	↓
LGR5	ONC	↑
LIFR	TS	↑
LPIN1	NC	↓
MAPKAPK3	NC	↑
MARVELD2	ONC	↑
MGAT5	ONC	↑
MLH1	TS	↑
MME	ONC	↓
MPP1	NC	↓
MTOR	ONC	↑
NEK6	ONC	↑
NGFR	TS	↑
NQO1*	ONC/TS	↓
OAS1A	NC	↑
OCLN	NC	↑
PDCD4*	ONC/TS	↑
PER2	TS	↓
PGLYRP2	NC	↑
PIGP	NC	↓
PML*	ONC/TS	↑
PPP1R1B	ONC	↓
PRKCZ	NC	↑
PXMP2	NC	↓
RASSF5	TS	↑
RFX5	NC	↑
SCAP	NC	↑
SELP	NC	↑
SERPINA1E	NC	↑
SERPINB1A	NC	↓
SERPINE1	ONC	↑
SFTPA1	ONC	↑
SIGLEC1	NC	↑

Table S3. – Classification of 124 genes of interest as ONC, D or TS (4/4).

**Genes deregulated in LImir21KO targeted by miR-21** *(predicted and validated)*	**Genes classified as** *Sources: CancerMine*	**Expression in LImiR-21KO transcriptomic** (p-value<0.05)
SLC16A13	NC	↓
SLC16A7	ONC	↓
SLC20A1	NC	↑
SLC37A1	NC	↑
SLC43A2	NC	↑
SQLE	ONC	↑
TAP1	NC	↑
TFF3	ONC	↑
TIRAP	NC	↑
TK1	NC	↓
TLR2	ONC	↑
TNFRSF14	TS	↑
TNFSF10	NC	↑
TNS1	ONC	↓

Table S4. – Gene Ontology enrichment analysis of genes unclassified by CancerMine

Functional Category (FDR<0.05)	Genes in list
Defense response	OAS1 CXCL9 PGLYRP2 TIRAP SELP FGB C6 PRKCZ CD5L DDX60 IL22RA1 C8A APPL1 TAP1 SERPINA1 SIGLEC1
Immune response	CXCL9 TIRAP C8A FGB C6 PRKCZ CD5L OAS1 SCAP TNFSF10 DDX60 APPL1 PGLYRP2 TAP1 FUCA2 ACP3 SERPINA1
Interspecies interaction between organisms	SIGLEC1 OAS1 CXCL9 PGLYRP2 TIRAP SELP DDX60 FGB C6 MAPKAPK3 IL22RA1 SLC20A1 C8A APPL1 TAP1 FUCA2
Pattern recognition receptor signaling pathway	GRAMD4 MAPKAPK3 TIRAP APPL1 DDX60 FGB
Immune effector process	OAS1 C8A C6 PRKCZ CD5L DDX60 CXCL9 APPL1 PGLYRP2 FUCA2 ACP3 SERPINA1
Immune system process	OAS1 CXCL9 TAP1 TIRAP C8A FGB C6 PRKCZ CD5L SCAP TNFSF10 DDX60 APPL1 PGLYRP2 SELP FUCA2 ACP3 SERPINA1
Response to external biotic stimulus	OAS1 CXCL9 PGLYRP2 TIRAP SELP DDX60 FGB C6 MAPKAPK3 IL22RA1 C8A APPL1 FUCA2
Response to other organism	OAS1 CXCL9 PGLYRP2 TIRAP SELP DDX60 FGB C6 MAPKAPK3 IL22RA1 C8A APPL1 FUCA2
Response to biotic stimulus	OAS1 CXCL9 PGLYRP2 TIRAP SELP DDX60 FGB C6 MAPKAPK3 IL22RA1 C8A APPL1 FUCA2
Inflammatory response	CXCL9 PRKCZ CD5L TIRAP APPL1 PGLYRP2 SELP SERPINA1 SIGLEC1
Ceramide translocation	ABCB1 ABCA2
Toll-like receptor signaling pathway	GRAMD4 MAPKAPK3 TIRAP APPL1 FGB
Positive regulation of response to stimulus	SLC20A1 TNFSF10 C8A FGB SELP C6 PRKCZ CD5L APPL1 EFNA1 ACP3 DDX60 TIRAP OCLN MAPKAPK3
Response to stress	ARHGEF10L OAS1 CXCL9 PGLYRP2 FGB TIRAP SELP SCAP C6 PRKCZ CD5L DDX60 IL22RA1 C8A APPL1 TAP1 SERPINA1 ABCB1 OCLN SIGLEC1
Humoral immune response	CXCL9 C8A FGB C6 CD5L PGLYRP2
Regulation of immune effector process	PRKCZ CD5L APPL1 PGLYRP2 DDX60 C6 C8A
Defense response to other organism	OAS1 PGLYRP2 TIRAP SELP FGB C6 DDX60 CXCL9 IL22RA1 C8A APPL1
Response to bacterium	CXCL9 PGLYRP2 SELP TIRAP FGB MAPKAPK3 IL22RA1 FUCA2
Sphingolipid translocation	ABCB1 ABCA2
Regulation of response to stimulus	SLC20A1 GRAMD4 TNFSF10 TIRAP C8A FGB SELP C6 PRKCZ CD5L APPL1 PGLYRP2 EFNA1 ACP3 ABCB1 DDX60 OCLN ARAP2 MAPKAPK3
Regulation of pattern recognition receptor signaling pathway	GRAMD4 APPL1 DDX60 TIRAP
Positive regulation of intracellular signal transduction	SLC20A1 TNFSF10 FGB SELP PRKCZ EFNA1 DDX60 TIRAP MAPKAPK3
Macropinocytosis	MAPKAPK3 APPL1
Regulation of complement activation	CD5L C6 C8A
Defense response to bacterium	PGLYRP2 SELP FGB IL22RA1 TIRAP
Vesicle-mediated transport	SIGLEC1 ABCA2 FGB CD5L MAPKAPK3 APPL1 SCAP FUCA2 ACP3 TAP1 SELP SERPINA1
SREBP signaling pathway	ARHGEF10L SCAP
Phosphate ion transmembrane transport	SLC20A1 SLC37A1
Ceramide transport	ABCB1 ABCA2
Cellular response to sterol depletion	ARHGEF10L SCAP

Genes deregulated in the transcriptomic analysis performed on hepatic tissues of LmiR21KO (Figure 1) were classified as oncogenes, tumor supressors, drivers and unclassified genes (Table S3) based on literature screening (source: CancerMine database). The list of unclassified genes from Table S3 was taken and Gene Ontology (GO) enrichment analysis based on Biological processes was performed on these genes using ShinyGO software. The enriched functional categories are represented on the left column and the genes involved in the category are indicated on the right column. Only functional categories with False Discovery Rate (FDR) < 5% are represented
